# Supplementary material for: Dynamics of collective cooperation under personalised strategy updates
Source: Nat Commun. 2024 Apr 11;15:3125. doi: 10.1038/s41467-024-47380-8 (PMC11006938; doi:10.1038/s41467-024-47380-8)
Supplement: Supplementary file 1 — Supplementary Information [file 41467_2024_47380_MOESM1_ESM.pdf]

Supplementary Information for

Dynamics of collective cooperation under personalised strategy updates

Yao Meng, Sean P. Cornelius, Yang-Yu Liu & Aming Li

Supplementary Note 1: Fixation probability at arbitrary update rate

We present the results on unweighted networks in main text. To show the generality of our framework, here we derive the theoretical results on weighted networks, where edge weights $w_{ij} = w_{ji}$ denote the interaction intensity between nodes i and j. And the weighted degree of node i is defined by $w_i = \sum_{j=1}^{N} w_{ij}$. The probability of a single step of random walk from node i to j is $p_{ij} = w_{ij}/w_i$. $p_{ij}^{(n)}$ represents the probability of an n-step random walk from node i to j and $\lim_{n\to\infty} p_{ij}^{(n)} = w_j/W$ [1], where $W = \sum_{i=1}^{N} w_i$ is the total weights of the network.

Individuals choose either cooperation (C) or defection (D) in the evolutionary game process. The state of the evolutionary process is thus given by $\mathbf{x} \in \{0,1\}^N$, where $x_i = 1$ indicates that the individual i chooses cooperation, otherwise $x_i = 0$ indicates defection for i. Evolutionary process is modelled by an evolutionary Markov chain $\mathbf{X}(t)$, where the state is updated through replacement events, which occur as Poisson processes [1]. For each individual i, the events of strategy updating follow the Poisson process at an arbitrary rate $\lambda_i > 0$. When i updates its strategy, the probability of imitating a randomly selected neighbour j's strategy is proportional to $w_{ij}F_j(\mathbf{x})$, where $F_j(\mathbf{x})$ is the fitness of individual j in state $\mathbf{x}$. Therefore, the probability for j transmitting its strategy to i in state $\mathbf{x}$ is

$$r_{ji}(\mathbf{x}) = \frac{\lambda_i}{\Lambda} \frac{w_{ij}F_j(\mathbf{x})}{\sum_{l=1}^{N} w_{il}F_l(\mathbf{x})}. \tag{1}$$

where $\Lambda = \sum_{i=1}^{N} \lambda_i$ represents the total rate of strategy updating in population.

In each round of the game, individuals interact pairwise with their neighbours and gain corresponding payoffs. For donation game [2], a cooperator pays a cost c and provides benefit b to the opponent, while defectors do not pay any cost or provide any benefit. The corresponding payoff ma-

trix is given in the Methods section in the main text. Then the average weighted payoff for individual i in state $\mathbf{x}$ is

$$\begin{aligned} f_i(\mathbf{x}) &= \sum_{l=1}^{N} p_{il} \left((b-c)x_i x_l - c x_i(1-x_l) + b(1-x_i)x_l\right) \\ &= -c x_i + b \sum_{l=1}^{N} p_{il} x_l. \end{aligned}$$

We first calculate the reproductive value π_i of individual i [3, 4, 5, 6], which is the unique solution of equations capturing that the expected reproductive value of new copies produced by i is equal to the corresponding expected loss

$$\begin{aligned} \sum_{j=1}^{N} r_{ij}^{\circ} \pi_j &= \sum_{j=1}^{N} r_{ji}^{\circ} \pi_i, \\ \sum_{i=1}^{N} \pi_i &= 1, \end{aligned} \tag{2}$$

where r_{ij}° denotes the probability of i replacing j in the case of neutral drift (the intensity of selection $\delta = 0$). We will still use the superscript $^{\circ}$ to denote the quantities under neutral drift. According to Equation (1), $r_{ij}^{\circ} = \lambda_j p_{ji}/\Lambda$. The reproductive value π_i is given by

$$\pi_i = \frac{w_i}{\lambda_i \sum_{l=1}^{N} \frac{w_l}{\lambda_l}}.$$

Then we obtain the reproductive-value-weighted birth rate of individual i which indicates the expected reproductive value of new copies produced by i

$$\widehat{b}_i(\mathbf{x}) = \sum_{j=1}^{N} r_{ij}(\mathbf{x}) \pi_j.$$

Similarly, the corresponding death rate of individual i capturing the expected loss of reproductive value is

$$\widehat{d}_i(\mathbf{x}) = \sum_{j=1}^{N} r_{ji}(\mathbf{x}) \pi_i.$$

Hence the reproductive-value-weighted frequency change of cooperation is [5, 6, 7]

$$\widehat{\Delta}(\mathbf{x}) := \sum_{i=1}^{N} x_i \left(\widehat{b}_i(\mathbf{x}) - \widehat{d}_i(\mathbf{x})\right) = \sum_{i=1}^{N} \pi_i \sum_{j=1}^{N} (x_j - x_i)\, r_{ji}(\mathbf{x}). \tag{3}$$

According to Equation (2), we have $\widehat{b}_i^{\circ} = \widehat{d}_i^{\circ}$. Thus under neutral drift, the reproductive-value-weighted frequency change $\widehat{\Delta}(\mathbf{x})$ is 0 for any state $\mathbf{x} \in \{0,1\}^N$. The fixation probability of cooperation

under mutant-appearance distribution of a single cooperator μ_C is [6]

$$\mathbb{E}_{\mu_C}[\rho_C] = \mathbb{E}^{\circ}_{\mu_C}[\widehat{\xi}] + \delta \mathbb{E}^{\circ}_{\mu_C}\left[\left\langle \left.\frac{\mathrm{d}\widehat{\Delta}(\mathbf{x})}{\mathrm{d}\delta}\right|_{\delta=0}\right\rangle^{\circ}_{\boldsymbol{\xi}}\right] + \mathcal{O}\left(\delta^2\right),$$

where $\boldsymbol{\xi} \in \{0,1\}^N$ indicates the initial state and $\widehat{\xi}$ is the reproductive-value-weighted frequency of cooperation at the initial state. Here $\langle \varphi \rangle^{\circ}_{\boldsymbol{\xi}}$ is defined as $\langle \varphi(\mathbf{x}) \rangle^{\circ}_{\boldsymbol{\xi}} = \sum_{t=0}^{\infty} \sum_{\mathbf{x}\in\{0,1\}^N} \mathbb{P}^{\circ}_{\boldsymbol{\xi}}\left[\mathbf{X}(t) = \mathbf{x}\right] \varphi(\mathbf{x})$ with $\varphi(\mathbf{1}) = \varphi(\mathbf{0}) = 0$, where $\mathbb{P}^{\circ}_{\boldsymbol{\xi}}\left[\mathbf{X}(t) = \mathbf{x}\right]$ indicates the neutral probability of the system reaching state $\mathbf{x}$ at time step t starting from $\boldsymbol{\xi}$ [6]. Note that here $\mathbf{1}$ represents the all cooperation state, and $\mathbf{0}$ indicates the all defection state. According to Equation (3), we have

$$\left.\frac{\mathrm{d}\widehat{\Delta}(\mathbf{x})}{\mathrm{d}\delta}\right|_{\delta=0} = \sum_{i=1}^{N} \pi_i \sum_{j=1}^{N} (x_j - x_i) \left.\frac{\mathrm{d}r_{ji}(\mathbf{x})}{\mathrm{d}\delta}\right|_{\delta=0},$$

where $\left.\frac{\mathrm{d}r_{ji}(\mathbf{x})}{\mathrm{d}\delta}\right|_{\delta=0} = \frac{\lambda_i}{\Lambda} p_{ij}(f_j(\mathbf{x}) - \sum_{l=1}^{N} p_{il} f_l(\mathbf{x}))$. Thus $\left.\frac{\mathrm{d}\widehat{\Delta}(\mathbf{x})}{\mathrm{d}\delta}\right|_{\delta=0}$ is given by

$$\begin{aligned}
\left.\frac{\mathrm{d}\widehat{\Delta}(\mathbf{x})}{\mathrm{d}\delta}\right|_{\delta=0} &= \frac{1}{\Lambda \sum_{i=1}^{N} \frac{w_i}{\lambda_i}} \sum_{i=1}^{N} \sum_{j=1}^{N} (x_j - x_i) w_i p_{ij} \left(f_j(\mathbf{x}) - \sum_{l=1}^{N} p_{il} f_l(\mathbf{x}) \right) \\
&= \frac{1}{\Lambda \sum_{i=1}^{N} \frac{w_i}{\lambda_i}} \left(\sum_{i=1}^{N} w_i x_i f_i(\mathbf{x}) - \sum_{i,j=1}^{N} w_i p_{ij}^{(2)} x_i f_j(\mathbf{x}) \right) \\
&= \frac{1}{\Lambda \sum_{i=1}^{N} \frac{w_i}{\lambda_i}} \left\{ c \left[\sum_{i=1}^{N} w_i (\widehat{x} - x_i^2) - \sum_{i,j=1}^{N} w_i p_{ij}^{(2)} (\widehat{x} - x_i x_j) \right] \right. \\
&\quad \left. -b \left[\sum_{i,j=1}^{N} w_i p_{ij} (\widehat{x} - x_i x_j) - \sum_{i,j=1}^{N} w_i p_{ij}^{(3)} (\widehat{x} - x_i x_j) \right] \right\},
\end{aligned}$$

where $\widehat{x}$ is the reproductive-value-weighted frequency of cooperation in state $\mathbf{x}$.

By defining $\eta_{ij}^{\mu_C} := \mathbb{E}^{\circ}_{\mu_C}[\langle \widehat{x} - x_i x_j \rangle^{\circ}_{\boldsymbol{\xi}}]$, we have the exact formula of fixation probability

$$\begin{aligned}
\mathbb{E}_{\mu_C}[\rho_C] =& \mathbb{E}^{\circ}_{\mu_C}[\widehat{\xi}] + \frac{\delta}{\Lambda \sum_{i=1}^{N} \frac{w_i}{\lambda_i}} \left[c \left(\sum_{i=1}^{N} w_i \eta_{ii}^{\mu_C} - \sum_{i,j=1}^{N} w_i p_{ij}^{(2)} \eta_{ij}^{\mu_C} \right) \right. \\
& \left. -b \left(\sum_{i,j=1}^{N} w_i p_{ij} \eta_{ij}^{\mu_C} - \sum_{i,j=1}^{N} w_i p_{ij}^{(3)} \eta_{ij}^{\mu_C} \right) \right] + O\left(\delta^2\right).
\end{aligned}$$

Hence the condition for selection favouring cooperation is

$$b/c > \frac{\sum_{i,j=1}^{N} w_i p_{ij}^{(2)} \eta_{ij}^{\mu_C} - \sum_{i=1}^{N} w_i \eta_{ii}^{\mu_C}}{\sum_{i,j=1}^{N} w_i p_{ij}^{(3)} \eta_{ij}^{\mu_C} - \sum_{i,j=1}^{N} w_i p_{ij} \eta_{ij}^{\mu_C}},$$

under which the fixation probability of cooperation exceeds that in the neutral case. $\eta_{ij}^{\mu_C}$ is the unique solution of the equations [6]

$$\eta_{ij}^{\mu_C} = \mathbb{E}_{\mu_C}^{\circ}[\widehat{\xi} - \xi_i \xi_j] + \sum_{l,k=1,k\neq i,j}^{N} r_{lk}^{\circ} \eta_{ij}^{\mu_C} + \sum_{l=1}^{N} r_{li}^{\circ} \eta_{lj}^{\mu_C} + \sum_{l=1}^{N} r_{lj}^{\circ} \eta_{li}^{\mu_C},$$

$$\sum_{i=1}^{N} \pi_i \eta_{ii}^{\mu_C} = 0.$$

We now offer the condition under which the selection favours cooperation over defection, namely $\mathbb{E}_{\mu_C}[\rho_C] > \mathbb{E}_{\mu_D}[\rho_D]$, where μ_D refers to the mutant-appearance distribution of the single defector at the initial state full of cooperators. Under uniform initialisation of the appearance of a single cooperator (defector), we have

$$\mathbb{E}_{\mu_C}[\xi_i] = \frac{1}{N} \quad \text{and} \quad \mathbb{E}_{\mu_D}[\xi_i] = \frac{N-1}{N}$$

for all $i \in \{1, ..., N\}$. Therefore,

$$\mathbb{E}_{\mu_C}^{\circ}[\widehat{\xi} - \xi_i \xi_j] = \mathbb{E}_{\mu_D}^{\circ}[\widehat{\xi} - \xi_i \xi_j] = \frac{1}{N}$$

if $i \neq j$ and

$$\mathbb{E}_{\mu_C}^{\circ}[\widehat{\xi} - \xi_i] = \mathbb{E}_{\mu_D}^{\circ}[\widehat{\xi} - \xi_i] = 0.$$

As we know

$$\left.\frac{\mathrm{d}\mathbb{E}_{\mu_D}[\rho_D]}{\mathrm{d}\delta}\right|_{\delta=0} = -\left.\frac{\mathrm{d}\mathbb{E}_{\mu_D}[\rho_C]}{\mathrm{d}\delta}\right|_{\delta=0}$$

according to $\mathbb{E}_{\mu_D}[\rho_C] = 1 - \mathbb{E}_{\mu_D}[\rho_D]$, the fixation probability of defection is

$$\mathbb{E}_{\mu_D}[\rho_D] = \frac{1}{N} - \frac{\delta}{\Lambda \sum_{i=1}^{N} \frac{w_i}{\lambda_i}} \left[c \left(\sum_{i=1}^{N} w_i \eta_{ii}^{\mu_D} - \sum_{i,j=1}^{N} w_i p_{ij}^{(2)} \eta_{ij}^{\mu_D} \right) \right.$$
$$\left. - b \left(\sum_{i,j=1}^{N} w_i p_{ij} \eta_{ij}^{\mu_D} - \sum_{i,j=1}^{N} w_i p_{ij}^{(3)} \eta_{ij}^{\mu_D} \right) \right] + O\left(\delta^2\right),$$

where the recurrence of $\eta_{ij}^{\mu_D}$ is

$$\eta_{ij}^{\mu_D} = \mathbb{E}_{\mu_D}^{\circ}[\widehat{\xi} - \xi_i\xi_j] + \sum_{l,k=1,k\neq i,j}^{N} r_{lk}^{\circ}\eta_{ij}^{\mu_D} + \sum_{l=1}^{N} r_{li}^{\circ}\eta_{lj}^{\mu_D} + \sum_{l=1}^{N} r_{lj}^{\circ}\eta_{li}^{\mu_D},$$
$$\sum_{i=1}^{N}\pi_i\eta_{ii}^{\mu_D} = 0.$$

We calculate the difference between fixation probability of cooperation and defection

$$\begin{aligned}\mathbb{E}_{\mu_C}[\rho_C] - \mathbb{E}_{\mu_D}[\rho_D] =& \frac{\delta}{\Lambda\sum_{i=1}^{N}\frac{w_i}{\lambda_i}}\left\{c\left[\sum_{i=1}^{N} w_i(\eta_{ii}^{\mu_C} + \eta_{ii}^{\mu_D}) - \sum_{i,j=1}^{N} w_i p_{ij}^{(2)}(\eta_{ij}^{\mu_C} + \eta_{ij}^{\mu_D})\right]\right.\\ &\left. - b\left[\sum_{i,j=1}^{N} w_i p_{ij}(\eta_{ij}^{\mu_C} + \eta_{ij}^{\mu_D}) - \sum_{i,j=1}^{N} w_i p_{ij}^{(3)}(\eta_{ij}^{\mu_C} + \eta_{ij}^{\mu_D})\right]\right\} + O\left(\delta^2\right),\end{aligned}$$

and we define $\eta_{ij}^{\mu_{CD}} = \eta_{ij}^{\mu_C} + \eta_{ij}^{\mu_D}$, which is given by the unique solution of equations

$$\eta_{ij}^{\mu_{CD}} = \mathbb{E}_{\mu_D}^{\circ}[\widehat{\xi} - \xi_i\xi_j] + \mathbb{E}_{\mu_C}^{\circ}[\widehat{\xi} - \xi_i\xi_j] + \sum_{l,k=1,k\neq i,j}^{N} r_{lk}^{\circ}\eta_{ij}^{\mu_{CD}} + \sum_{l=1}^{N} r_{li}^{\circ}\eta_{lj}^{\mu_{CD}} + \sum_{l=1}^{N} r_{lj}^{\circ}\eta_{li}^{\mu_{CD}},$$
$$\sum_{i=1}^{N}\pi_i\eta_{ii}^{\mu_{CD}} = 0.$$

Letting $\mathbb{E}_{\mu_C}[\rho_C] - \mathbb{E}_{\mu_D}[\rho_D] > 0$, the success condition for selection favouring cooperation over defection is

$$b/c > \frac{\sum_{i,j=1}^{N} w_i p_{ij}^{(2)}\eta_{ij}^{\mu_{CD}} - \sum_{i=1}^{N} w_i\eta_{ii}^{\mu_{CD}}}{\sum_{i,j=1}^{N} w_i p_{ij}^{(3)}\eta_{ij}^{\mu_{CD}} - \sum_{i,j=1}^{N} w_i p_{ij}\eta_{ij}^{\mu_{CD}}},$$

where $\eta_{ij}^{\mu_{CD}} = 2\eta_{ij}^{\mu_C}$. The unique solution of $\eta_{ij}^{\mu_C}$ is

$$\eta_{ij}^{\mu_C} = \begin{cases}\frac{\Lambda}{N(\lambda_i+\lambda_j)} + \sum_{k=1}^{N}\frac{\lambda_i}{\lambda_i+\lambda_j}p_{ik}\eta_{kj}^{\mu_C} + \sum_{k=1}^{N}\frac{\lambda_j}{\lambda_i+\lambda_j}p_{jk}\eta_{ki}^{\mu_C} & \text{if } \ i\neq j\\ 0 & \text{if } \ i=j\end{cases}. \tag{4}$$

In this case, the condition for $\mathbb{E}_{\mu_C}[\rho_C] > \mathbb{E}_{\mu_D}[\rho_D]$ and $\mathbb{E}_{\mu_C}[\rho_C] > 1/N$ is the same, and the critical benefit-to-cost ratio is

$$C^* = \frac{\sum_{i,j=1}^{N} w_i p_{ij}^{(2)}\eta_{ij}^{\mu_C}}{\sum_{i,j=1}^{N} w_i p_{ij}^{(3)}\eta_{ij}^{\mu_C} - \sum_{i,j=1}^{N} w_i p_{ij}\eta_{ij}^{\mu_C}}. \tag{5}$$

The fixation probability of cooperation with arbitrary rate of strategy updating under uniform ini-

tialisation is

$$\mathbb{E}_{\mu_C}[\rho_C] = \frac{1}{N} + \frac{\delta}{\Lambda \sum_{i=1}^{N} \frac{w_i}{\lambda_i}} \left[-c \sum_{i,j=1}^{N} w_i p_{ij}^{(2)} \eta_{ij}^{\mu_C} + b \left(\sum_{i,j=1}^{N} w_i p_{ij}^{(3)} \eta_{ij}^{\mu_C} - \sum_{i,j=1}^{N} w_i p_{ij} \eta_{ij}^{\mu_C} \right) \right] + O\left(\delta^2\right).$$

Supplementary Note 2: Intuitive explanation

To provide an intuitive explanation of the underlying mechanism of the effect of update rates, we use the pair approximation and the diffusion approximation to describe the dynamical evolutionary process. We first define the probability of finding an individual with strategy Y next to an individual with strategy X as ϕ_{XY}, where $\mathrm{X}, \mathrm{Y} \in \{\mathrm{C}, \mathrm{D}\}$. Then, the marginal probability ϕ_{X} is given by $\phi_{\mathrm{X}} = \phi_{\mathrm{XC}} + \phi_{\mathrm{XD}}$, which represents the probability that an edge starts from an individual with strategy X. We further define $q_{\mathrm{X|Y}} = \phi_{\mathrm{YX}}/\phi_{\mathrm{Y}}$ as the conditional probability that a neighbour of a node with strategy Y takes the strategy X. And the probability that a node with degree k has the strategy C (D) is represented by $\phi_{k,\mathrm{C}}$ ($\phi_{k,\mathrm{D}}$). The degree distribution of the network is captured by $p(k)$, which represents the probability of a node with k neighbours.

2.1 Pair approximation

We consider the case that individuals with k neighbours have the update rate $\lambda(k)$, and the probability that an individual with k neighbours is randomly chosen for updating is $\lambda(k)Np(k)/\Lambda$. Here $\Lambda = \sum_{k=1}^{N-1} Np(k)\lambda(k)$ represents the total rate of strategy updating in population. Specifically, the probability that a defector with k_{C} cooperative neighbours and k_{D} defective neighbours ($k_{\mathrm{C}}+k_{\mathrm{D}} = k$) is $(k!/k_{\mathrm{C}}!k_{\mathrm{D}}!)q_{\mathrm{C|D}}^{k_{\mathrm{C}}} q_{\mathrm{D|D}}^{k_{\mathrm{D}}}$. Let $f_{\mathrm{C}} = 1+\delta h_{\mathrm{C}}$ denote the fitness of a cooperative neighbour of this defector and $f_{\mathrm{D}} = 1+\delta h_{\mathrm{D}}$ denote the fitness of a defective neighbour of this defector, where h_{C} (h_{D}) captures the expected payoff of each cooperative (defective) neighbour. Therefore, ϕ_{C} increase by $k/\langle k\rangle N$ with probability

$$\begin{aligned}
\mathrm{Prob}\left(\Delta\phi_{\mathrm{C}} = \frac{k}{\langle k\rangle N}\right) &= \frac{\lambda(k)N}{\Lambda} p(k)\phi_{k,\mathrm{D}} \sum_{k_{\mathrm{C}}+k_{\mathrm{D}}=k} \frac{k!}{k_{\mathrm{C}}!k_{\mathrm{D}}!} q_{\mathrm{C|D}}^{k_{\mathrm{C}}} q_{\mathrm{D|D}}^{k_{\mathrm{D}}} \frac{k_{\mathrm{C}} f_{\mathrm{C}}}{k_{\mathrm{C}} f_{\mathrm{C}} + k_{\mathrm{D}} f_{\mathrm{D}}} \\
&= \frac{\lambda(k)N}{\Lambda} p(k)\phi_{k,\mathrm{D}} \left[q_{\mathrm{C|D}} + \delta q_{\mathrm{C|D}} q_{\mathrm{D|D}} (1 - \frac{1}{k})(h_{\mathrm{C}} - h_{\mathrm{D}}) \right] + O\left(\delta^2\right).
\end{aligned}$$

Regarding pairs, the number of CC-pairs increases by $2k_{\rm C}/\langle k\rangle N$ with probability

$$\begin{aligned}\text{Prob}\left(\Delta\phi_{\rm CC}=\frac{2k_{\rm C}}{\langle k\rangle N}\right)&=\sum_{k\geq k_{\rm C}}\frac{\lambda(k)N}{\Lambda}p(k)\phi_{k,{\rm D}}\frac{k!}{k_{\rm C}!k_{\rm D}!}q_{\rm C|D}^{k_{\rm C}}q_{\rm D|D}^{k_{\rm D}}\frac{k_{\rm C}f_{\rm C}}{k_{\rm C}f_{\rm C}+k_{\rm D}f_{\rm D}}\\&=\sum_{k\geq k_{\rm C}}\frac{\lambda(k)N}{\Lambda}p(k)\phi_{k,{\rm D}}\frac{k!}{k_{\rm C}!k_{\rm D}!}q_{\rm C|D}^{k_{\rm C}}q_{\rm D|D}^{k_{\rm D}}\frac{k_{\rm C}}{k}+O\left(\delta\right).\end{aligned}$$

Analogously, for a cooperator with k neighbours is randomly chosen for updating, we define $g_{\rm C}=1+\delta l_{\rm C}$ as the fitness of a cooperative neighbour of this cooperator, and the fitness of a defective neighbour of this cooperator is denoted by $g_{\rm D}=1+\delta l_{\rm D}$, where $l_{\rm C}$ ($l_{\rm D}$) captures the expected payoff of each cooperative (defective) neighbour. We have

$$\begin{aligned}\text{Prob}\left(\Delta\phi_{\rm C}=-\frac{k}{\langle k\rangle N}\right)&=\frac{\lambda(k)N}{\Lambda}p(k)\phi_{k,{\rm C}}\sum_{k_{\rm C}+k_{\rm D}=k}\frac{k!}{k_{\rm C}!k_{\rm D}!}q_{\rm C|C}^{k_{\rm C}}q_{\rm D|C}^{k_{\rm D}}\frac{k_{\rm D}g_{\rm D}}{k_{\rm C}g_{\rm C}+k_{\rm D}g_{\rm D}}\\&=\frac{\lambda(k)N}{\Lambda}p(k)\phi_{k,{\rm C}}\left[q_{\rm D|C}+\delta q_{\rm C|C}q_{\rm D|C}(1-\frac{1}{k})(l_{\rm D}-l_{\rm C})\right]+O\left(\delta^2\right).\end{aligned}$$

and

$$\begin{aligned}\text{Prob}\left(\Delta\phi_{\rm CC}=-\frac{2k_{\rm C}}{\langle k\rangle N}\right)&=\sum_{k\geq k_{\rm C}}\frac{\lambda(k)N}{\Lambda}p(k)\phi_{k,{\rm C}}\frac{k!}{k_{\rm C}!k_{\rm D}!}q_{\rm C|C}^{k_{\rm C}}q_{\rm D|C}^{k_{\rm D}}\frac{k_{\rm D}g_{\rm D}}{k_{\rm C}g_{\rm C}+k_{\rm D}g_{\rm D}}\\&=\sum_{k\geq k_{\rm C}}\frac{\lambda(k)N}{\Lambda}p(k)\phi_{k,{\rm C}}\frac{k!}{k_{\rm C}!k_{\rm D}!}q_{\rm C|C}^{k_{\rm C}}q_{\rm D|C}^{k_{\rm D}}\frac{k_{\rm D}}{k}+O\left(\delta\right).\end{aligned}$$

2.2 Diffusion approximation

The time derivatives of $\phi_{\rm C}$ and $\phi_{\rm CC}$ are given by

$$\begin{aligned}\frac{{\rm d}\phi_{\rm C}}{{\rm d}t}&=\sum_{k=1}^{N-1}\frac{k}{\langle k\rangle N}\text{Prob}\left(\Delta\phi_{\rm C}=\frac{k}{\langle k\rangle N}\right)+\sum_{k=1}^{N-1}\left(-\frac{k}{\langle k\rangle N}\right)\text{Prob}\left(\Delta\phi_{\rm C}=-\frac{k}{\langle k\rangle N}\right)\\&=\delta\sum_{k=1}^{N-1}\frac{\lambda(k)}{\langle k\rangle\Lambda}p(k)(k-1)\left[(h_{\rm C}-h_{\rm D})\phi_{k,{\rm D}}q_{\rm C|D}q_{\rm D|D}-(l_{\rm D}-l_{\rm C})\phi_{k,{\rm C}}q_{\rm D|C}q_{\rm C|C}\right]\\&+\sum_{k=1}^{N-1}\frac{k\lambda(k)p(k)\phi_{\rm CD}}{\langle k\rangle\Lambda\phi_{\rm C}\phi_{\rm D}}(\phi_{\rm C}-\phi_{k,{\rm C}})+O\left(\delta^2\right)\end{aligned}$$

and

$$\begin{aligned}\frac{{\rm d}\phi_{\rm CC}}{{\rm d}t}&=\sum_{k_{\rm C}=1}^{N-1}\frac{2k_{\rm C}}{\langle k\rangle N}\text{Prob}\left(\Delta\phi_{\rm CC}=\frac{2k_{\rm C}}{\langle k\rangle N}\right)+\sum_{k_{\rm C}=1}^{N-1}\left(-\frac{2k_{\rm C}}{\langle k\rangle N}\right)\text{Prob}\left(\Delta\phi_{\rm C}=-\frac{2k_{\rm C}}{\langle k\rangle N}\right)\\&=\sum_{k=1}^{N-1}\frac{2\lambda(k)p(k)}{\langle k\rangle\Lambda}\left[\phi_{k,{\rm D}}q_{\rm C|D}(kq_{\rm C|D}+q_{\rm D|D})-\phi_{k,{\rm C}}q_{\rm C|C}q_{\rm D|C}(k-1)\right]+O\left(\delta\right).\end{aligned}$$

Similarly, the time evolution of $\phi_{k,\mathrm{C}}$ is

$$\begin{aligned}\frac{\mathrm{d}\phi_{k,\mathrm{C}}}{\mathrm{d}t} &= \frac{1}{Np(k)}\mathrm{Prob}\left(\Delta\phi_{k,\mathrm{C}} = \frac{1}{Np(k)}\right) - \frac{1}{Np(k)}\mathrm{Prob}\left(\Delta\phi_{k,\mathrm{C}} = -\frac{1}{Np(k)}\right)\\ &= \frac{\lambda(k)\phi_{\mathrm{CD}}}{\Lambda\phi_{\mathrm{C}}\phi_{\mathrm{D}}}(\phi_{\mathrm{C}} - \phi_{k,\mathrm{C}}) + O(\delta).\end{aligned}$$

Here we consider the slow manifold of the dynamical system, where the constant terms of ϕ_{CC}, $\phi_{k,\mathrm{C}}$ and ϕ_{C} equal to 0. By letting the constant terms of $\mathrm{d}\phi_{k,\mathrm{C}}/\mathrm{d}t$ and $\mathrm{d}\phi_{\mathrm{C}}/\mathrm{d}t$ equal to 0, we have

$$\phi_{\mathrm{C}} - \phi_{k,\mathrm{C}} = 0.$$

By letting the constant term of $\mathrm{d}\phi_{\mathrm{CC}}/\mathrm{d}t$ equal to 0, we have

$$q_{\mathrm{C|C}} - q_{\mathrm{C|D}} = \frac{\sum_{k=1}^{N-1} p(k)\lambda(k)}{\sum_{k=1}^{N-1} p(k)\lambda(k)(k-1)} = \frac{1}{\sum_{k=1}^{N-1} p(k)\lambda(k)kN/\Lambda - 1}.$$

Our results recover the case with identical update rate ($\lambda_i = 1$), where

$$q_{\mathrm{C|C}} - q_{\mathrm{C|D}} = \frac{1}{\langle k\rangle - 1}$$

is obtained. When $\lambda_i = k_i$, the total rate is $\Lambda = \sum_{k=1}^{N-1} Np(k)k$, we have

$$q_{\mathrm{C|C}} - q_{\mathrm{C|D}} = \frac{1}{\langle k^2\rangle/\langle k\rangle - 1}.$$

Since $\langle k^2\rangle > \langle k\rangle^2$ for heterogeneous networks, $q_{\mathrm{C|C}} - q_{\mathrm{C|D}} < 1/(\langle k\rangle - 1)$. For the case of $\lambda_i = 1/k_i$ where the total rate is $\Lambda = \sum_{k=1}^{N-1} Np(k)/k$, we have

$$q_{\mathrm{C|C}} - q_{\mathrm{C|D}} = \frac{1}{N/\Lambda - 1} = \frac{1}{\frac{1}{\sum_{k=1}^{N-1} p(k)/k} - 1}.$$

For a wide range of heterogeneous networks including Erdös-Rényi, small-world and scale-free networks, $1/\sum_{k=1}^{N-1} p(k)/k < \langle k\rangle$. Therefore, we have $q_{\mathrm{C|C}} - q_{\mathrm{C|D}} > 1/(\langle k\rangle - 1)$ when $\lambda_i = 1/k_i$.

Supplementary Note 3: Theoretical approximation of C^*

In this section, we first offer the theoretical approximation for the critical threshold C^* for promoting cooperation with the random invasion of a single cooperator (defector). And then we introduce the

sufficient condition for relaxing C^* on large unweighted heterogeneous networks.

For simplification, we use η_{ij} to denote $\eta_{ij}^{\mu_C}$ in Equation (5). η_{ij} has the natural interpretation of the coalescence time starting from nodes i and j [8, 9, 10, 11]. We define $\eta^{(n)} = \frac{1}{W}\sum_{i,j=1}^{N} w_i p_{ij}^{(n)} \eta_{ij}$ as the weighted average coalescence time. Then we rewrite Equation (5) as

$$C^* = \frac{\eta^{(2)}}{\eta^{(3)} - \eta^{(1)}}. \tag{6}$$

According to Equation (4), we have the recurrence relation for $\eta^{(n)}$

$$\begin{aligned}
\eta^{(n)} &= \frac{1}{W}\sum_{i,j=1}^{N} w_i p_{ij}^{(n)} \eta_{ij} \\
&= \frac{1}{W}\sum_{i,j\in\{1,\ldots,N\}(i\neq j)} w_i p_{ij}^{(n)} \left[\frac{\Lambda}{N(\lambda_i+\lambda_j)} + \sum_{k=1}^{N}\frac{\lambda_i}{\lambda_i+\lambda_j} p_{ik}\eta_{kj} + \sum_{k=1}^{N}\frac{\lambda_j}{\lambda_i+\lambda_j} p_{jk}\eta_{ki}\right] \\
&= \sum_{i,j=1}^{N}\frac{w_i}{W} p_{ij}^{(n)} \frac{\Lambda}{N(\lambda_i+\lambda_j)} + \sum_{i,j,k=1}^{N}\frac{w_i}{W} p_{ij}^{(n)} \frac{2\lambda_j}{\lambda_i+\lambda_j} p_{jk}\eta_{ik} - \sum_{i=1}^{N}\frac{w_i}{W} p_{ii}^{(n)}\left(\frac{\Lambda}{2N\lambda_i} + \sum_{k=1}^{N} p_{ik}\eta_{ik}\right).
\end{aligned}$$

Here we define $\widetilde{\eta}^{(n+1)}$ by

$$\widetilde{\eta}^{(n+1)} = \sum_{i,j,k=1}^{N}\frac{w_i}{W} p_{ij}^{(n)} \frac{2\lambda_j}{\lambda_i+\lambda_j} p_{jk}\eta_{ik},$$

and define η_{ii}^{+} by

$$\eta_{ii}^{+} = \frac{\Lambda}{2N\lambda_i} + \sum_{k=1}^{N} p_{ik}\eta_{ik}. \tag{7}$$

Thus the recurrence relation between $\eta^{(n)}$ and $\widetilde{\eta}^{(n+1)}$ is

$$\eta^{(n)} = \sum_{i,j=1}^{N}\frac{w_i}{W} p_{ij}^{(n)} \frac{\Lambda}{N(\lambda_i+\lambda_j)} + \widetilde{\eta}^{(n+1)} - \sum_{i=1}^{N}\frac{w_i}{W} p_{ii}^{(n)} \eta_{ii}^{+}.$$

It should be noted that the network has no self-loops so that $p_{ii}^{(1)} = 0$ for all node i. For $n = 1, 2$, we have

$$\begin{aligned}
\eta^{(1)} &= \sum_{i,j=1}^{N}\frac{w_i}{W} p_{ij} \frac{\Lambda}{N(\lambda_i+\lambda_j)} + \widetilde{\eta}^{(2)}, \\
\eta^{(2)} &= \sum_{i,j=1}^{N}\frac{w_i}{W} p_{ij}^{(2)} \frac{\Lambda}{N(\lambda_i+\lambda_j)} + \widetilde{\eta}^{(3)} - \sum_{i=1}^{N}\frac{w_i}{W} p_{ii}^{(2)} \eta_{ii}^{+}.
\end{aligned} \tag{8}$$

By letting $n \to \infty$, we further obtain the relation between $\eta^{(\infty)}$ and $\widetilde{\eta}^{(\infty)}$

$$\eta^{(\infty)} = \frac{1}{W^2} \sum_{i,j=1}^{N} w_i w_j \frac{\Lambda}{N(\lambda_i + \lambda_j)} + \widetilde{\eta}^{(\infty)} - \frac{1}{W^2} \sum_{i=1}^{N} {w_i}^2 \eta_{ii}^{+}, \tag{9}$$

where $\eta^{(\infty)} = \frac{1}{W^2} \sum_{i,j=1}^{N} w_i w_j \eta_{ij}$ and $\widetilde{\eta}^{(\infty)} = \frac{1}{W^2} \sum_{i,j,k=1}^{N} w_i \frac{2\lambda_j}{\lambda_i + \lambda_j} w_{jk} \eta_{ik}$. After defining the difference $\Delta_{\widetilde{\eta}^{(n)}} = \widetilde{\eta}^{(n)} - \eta^{(n)}$, we have

$$\Delta_{\widetilde{\eta}^{(2)}} = \widetilde{\eta}^{(2)} - \eta^{(2)} = \sum_{i,j,k=1}^{N} \frac{w_i}{W} p_{ij} \frac{\lambda_j - \lambda_i}{\lambda_i + \lambda_j} p_{jk} \eta_{ik}, \tag{10}$$

$$\Delta_{\widetilde{\eta}^{(3)}} = \widetilde{\eta}^{(3)} - \eta^{(3)} = \sum_{i,j,k=1}^{N} \frac{w_i}{W} p_{ij}^{(2)} \frac{\lambda_j - \lambda_i}{\lambda_i + \lambda_j} p_{jk} \eta_{ik}, \tag{11}$$

$$\Delta_{\widetilde{\eta}^{(\infty)}} = \widetilde{\eta}^{(\infty)} - \eta^{(\infty)} = \frac{1}{W^2} \sum_{i,j,k=1}^{N} w_i \frac{\lambda_j - \lambda_i}{\lambda_i + \lambda_j} w_{jk} \eta_{ik}. \tag{12}$$

Together with Equation (8), we further obtain

$$\begin{aligned}
\eta^{(1)} &= \sum_{i,j=1}^{N} \frac{w_i}{W} p_{ij} \eta_{ij} = \sum_{i=1}^{N} \frac{w_i}{W} \eta_{ii}^{+} - \sum_{i=1}^{N} \frac{w_i}{W} \frac{\Lambda}{2N\lambda_i}, \\
\eta^{(2)} &= \widetilde{\eta}^{(2)} - \Delta_{\widetilde{\eta}^{(2)}} = \eta^{(1)} - \sum_{i,j=1}^{N} \frac{w_i}{W} p_{ij} \frac{\Lambda}{N(\lambda_i + \lambda_j)} - \Delta_{\widetilde{\eta}^{(2)}}, \\
\eta^{(3)} &= \widetilde{\eta}^{(3)} - \Delta_{\widetilde{\eta}^{(3)}} = \eta^{(2)} + \sum_{i=1}^{N} \frac{w_i}{W} p_{ii}^{(2)} \eta_{ii}^{+} - \sum_{i,j=1}^{N} \frac{w_i}{W} p_{ij}^{(2)} \frac{\Lambda}{N(\lambda_i + \lambda_j)} - \Delta_{\widetilde{\eta}^{(3)}}.
\end{aligned} \tag{13}$$

Substituting Equation (6) into Equation (13), we have the exact critical ratio

$$C^* = \frac{\sum_{i=1}^{N} \frac{w_i}{W} \eta_{ii}^{+} - \sum_{i=1}^{N} \frac{w_i}{W} \frac{\Lambda}{2N\lambda_i} - \sum_{i,j=1}^{N} \frac{w_i}{W} p_{ij} \frac{\Lambda}{N(\lambda_i+\lambda_j)} - \Delta_{\widetilde{\eta}^{(2)}}}{\sum_{i=1}^{N} \frac{w_i}{W} p_{ii}^{(2)} \eta_{ii}^{+} - \sum_{i,j=1}^{N} \frac{w_i}{W} (p_{ij} + p_{ij}^{(2)}) \frac{\Lambda}{N(\lambda_i+\lambda_j)} - \Delta_{\widetilde{\eta}^{(2)}} - \Delta_{\widetilde{\eta}^{(3)}}}. \tag{14}$$

The above expression still requires solving $N(N-1)/2$ linear equations. We now utilise the mean-field approximation approach [12] for achieving analytical results without solving linear equations. Here we replace η_{ii}^{+} by the average value $\overline{\eta}_{ii}^{+}$ in Equation (9), and we then obtain

$$\overline{\eta}_{ii}^{+} = \frac{W^2}{\sum_{i=1}^{N} w_i^2} \left(\sum_{i,j=1}^{N} \frac{w_i w_j \Lambda}{W^2 N(\lambda_i + \lambda_j)} + \Delta_{\widetilde{\eta}^{(\infty)}} \right). \tag{15}$$

Furthermore, we replace every η_{ij} $(i \neq j)$ by the average value $\overline{\eta}$ in Equations (7) and (12), then we have

$$\overline{\eta}_{ii}^{+} \approx \frac{1}{N}\sum_{i=1}^{N}\frac{\Lambda}{2N\lambda_i} + \overline{\eta}, \tag{16}$$

$$\Delta_{\widetilde{\eta}^{(\infty)}} \approx -\frac{\overline{\eta}}{W^2}\sum_{i,j=1}^{N} w_i \frac{\lambda_j - \lambda_i}{\lambda_i + \lambda_j} w_{ij} = -\frac{\overline{\eta}}{W^2}\sum_{i,j=1,i<j}^{N} (w_i - w_j)\frac{\lambda_j - \lambda_i}{\lambda_i + \lambda_j} w_{ij}. \tag{17}$$

Substituting Equation (15) into Equations (16) and (17), $\overline{\eta}$ is given by

$$\overline{\eta} = \frac{\frac{1}{\sum_{i=1}^{N} w_i^2}\sum_{i,j=1}^{N}\frac{w_i w_j \Lambda}{N(\lambda_i+\lambda_j)} - \frac{1}{N}\sum_{i=1}^{N}\frac{\Lambda}{2N\lambda_i}}{1 + \frac{1}{\sum_{i=1}^{N} w_i^2}\sum_{i,j=1}^{N} w_i \frac{\lambda_j-\lambda_i}{\lambda_i+\lambda_j} w_{ij}}.$$

We can also obtain $\Delta_{\widetilde{\eta}^{(2)}}$ and $\Delta_{\widetilde{\eta}^{(3)}}$ in Equations (10) and (11) as follows

$$\Delta_{\widetilde{\eta}^{(2)}} \approx -\frac{\overline{\eta}}{W}\sum_{i,j=1}^{N} w_i p_{ij}\frac{\lambda_j - \lambda_i}{\lambda_i + \lambda_j} p_{ji} = -\frac{\overline{\eta}}{W}\sum_{i,j=1,i<j}^{N} p_{ij}p_{ji}(w_i - w_j)\frac{\lambda_j - \lambda_i}{\lambda_i + \lambda_j}, \tag{18}$$

$$\Delta_{\widetilde{\eta}^{(3)}} \approx -\frac{\overline{\eta}}{W}\sum_{i,j=1}^{N} w_i p_{ij}^{(2)}\frac{\lambda_j - \lambda_i}{\lambda_i + \lambda_j} p_{ji} = -\frac{\overline{\eta}}{W}\sum_{i,j=1,i<j}^{N} p_{ij}^{(2)}p_{ji}(w_i - w_j)\frac{\lambda_j - \lambda_i}{\lambda_i + \lambda_j}. \tag{19}$$

By replacing η_{ii}^{+} with $\overline{\eta}_{ii}^{+}$ in Equation (14), the benefit-to-cost ratio is

$$C^* \approx \frac{\frac{1}{\sum_{i=1}^{N} w_i^2}\sum_{i,j=1}^{N}\frac{w_i w_j \Lambda}{N(\lambda_i+\lambda_j)} - \sum_{i=1}^{N}\frac{w_i \Lambda}{2WN\lambda_i} - \sum_{i,j=1}^{N}\frac{w_i}{W}p_{ij}\frac{\Lambda}{N(\lambda_i+\lambda_j)} - \Delta_{\widetilde{\eta}^{(2)}} + \frac{W^2}{\sum_{i=1}^{N} w_i^2}\Delta_{\widetilde{\eta}^{(\infty)}}}{\frac{\sum_{i=1}^{N} w_i p_{ii}^{(2)}}{W\sum_{i=1}^{N} w_i^2}\sum_{i,j=1}^{N}\frac{w_i w_j \Lambda}{N(\lambda_i+\lambda_j)} - \sum_{i,j=1}^{N}\frac{w_i}{W}(p_{ij} + p_{ij}^{(2)})\frac{\Lambda}{N(\lambda_i+\lambda_j)} - \Delta_{\widetilde{\eta}^{(2)}} - \Delta_{\widetilde{\eta}^{(3)}} + \frac{W\sum_{i=1}^{N} w_i p_{ii}^{(2)}}{\sum_{i=1}^{N} w_i^2}\Delta_{\widetilde{\eta}^{(\infty)}}}, \tag{20}$$

where $\Delta_{\widetilde{\eta}^{(\infty)}}$, $\Delta_{\widetilde{\eta}^{(2)}}$ and $\Delta_{\widetilde{\eta}^{(3)}}$ are given in Equations (17)–(19), respectively.

3.1 C^* for homogeneous networks

We consider the unweighted networks, where $w_{ij} = w_{ji} = 1$ if there is an edge between nodes i and j. Note that here $w_i = k_i$ on unweighted networks, where k_i is the degree of node i. We define $K = \sum_i k_i$ as the summation of all nodes' degree, and $\zeta = \sum_{i,j}\frac{k_i k_j \Lambda}{NK^2(\lambda_i+\lambda_j)}$.

For homogeneous networks, we have $\Delta_{\widetilde{\eta}^{(2)}} \approx \Delta_{\widetilde{\eta}^{(3)}} \approx \Delta_{\widetilde{\eta}^{(\infty)}} \approx 0$ according to Equations (17)–(19). Then, we have

$$C^* \approx \frac{N\zeta - \sum_{i=1}^{N}\frac{k}{K}\frac{\Lambda}{2N\lambda_i} - \sum_{i,j=1}^{N}\frac{k}{K}p_{ij}\frac{\Lambda}{N(\lambda_i+\lambda_j)}}{\frac{N}{k}\zeta - \sum_{i,j=1}^{N}\frac{k}{K}(p_{ij} + p_{ij}^{(2)})\frac{\Lambda}{N(\lambda_i+\lambda_j)}},$$

where k is the number of neighbours for each individual on homogeneous networks. By taking the

limit of large N in the above formula, we arrive at the famous simple rule $b/c > k$ reported in [13] on homogeneous networks with identical individual update rate. But note that here we show that the rule is also applicable for arbitrary individual update rate. This result indicates that the variations of update rates lead to negligible influence on the condition for favouring the emergence of cooperation when networks are sufficiently large.

3.2 C^* for heterogeneous networks

For heterogeneous networks, $\Delta_{\widetilde{\eta}^{(2)}}, \Delta_{\widetilde{\eta}^{(3)}}$ and $\Delta_{\widetilde{\eta}^{(\infty)}}$ are not 0. Here $\langle k \rangle$ indicates the average degree of the network. According to Equations (17)–(19), we obtain the relations

$$\Delta_{\widetilde{\eta}^{(2)}} \approx N\Delta_{\widetilde{\eta}^{(\infty)}}/\langle k \rangle \quad \text{and} \quad \Delta_{\widetilde{\eta}^{(3)}} \approx N\Delta_{\widetilde{\eta}^{(\infty)}}/\langle k \rangle^2.$$

And the critical benefit-to-cost ratio for large heterogeneous networks is then

$$C^* \approx \langle k \rangle \frac{\langle k \rangle^3 \zeta + (\langle k \rangle^3 - \langle k^2 \rangle)\Delta_{\widetilde{\eta}^{(\infty)}}}{\langle k \rangle^3 \zeta + (\langle k \rangle^3 - \langle k \rangle \langle k^2 \rangle - \langle k^2 \rangle)\Delta_{\widetilde{\eta}^{(\infty)}}}, \tag{21}$$

where $\langle k^2 \rangle$ represents the second moment of the degree distribution. We therefore observe that the quantity $\Delta_{\widetilde{\eta}^{(\infty)}}$ still remains to be a significant factor causing the deviations of the critical ratio from $\langle k \rangle$. This explains the large variations of the critical ratio over different update rates on heterogeneous networks numerically shown in Fig. 5a in the main text.

3.3 Sufficient condition for relaxing critical ratio

Here we provide a sufficient condition for reducing the critical ratio C^* on large unweighted heterogeneous networks.

Theorem 1. *For any $i, j \in \{1, ..., N\}$, if $k_j > k_i$ and $\lambda_j < \lambda_i$, then we have the critical ratio $C^* < k$ as $N \to \infty$. For any $i, j \in \{1, ..., N\}$, if $k_j > k_i$ and $\lambda_j > \lambda_i$, then we have the critical ratio $C^* > k$ as $N \to \infty$.*

This condition indicates that on large heterogeneous networks, if the update rates of individuals vary inversely to the nodes' degrees, the critical ratio C^* will be decreased below the average degree $\langle k \rangle$.

Proof. With the condition of $k_j > k_i$ and $\lambda_j < \lambda_i$ for any $i, j \in \{1, ..., N\}$, we have

$$\Delta_{\widetilde{\eta}(\infty)} \approx -\frac{\overline{\eta}}{K^2}\sum_{i,j=1}^{N} k_i \frac{\lambda_j - \lambda_i}{\lambda_i + \lambda_j} w_{ij} = -\frac{\overline{\eta}}{K^2}\sum_{i,j=1,i<j}^{N} (k_i - k_j)\frac{\lambda_j - \lambda_i}{\lambda_i + \lambda_j} w_{ij} < 0.$$

According to Equation (21), the critical ratio C^* is given by

$$\lim_{N\to\infty} C^* \approx \langle k\rangle + \frac{\langle k\rangle^2\langle k^2\rangle \Delta_{\widetilde{\eta}(\infty)}}{\langle k\rangle^3\zeta + (\langle k\rangle^3 - \langle k\rangle\langle k^2\rangle - \langle k^2\rangle)\Delta_{\widetilde{\eta}(\infty)}}.$$

When $\Delta_{\widetilde{\eta}(\infty)} < 0$, we get the conclusion that $\lim_{N\to\infty} C^* < \langle k\rangle$. Similarly, under the condition of $k_j > k_i$ and $\lambda_j > \lambda_i$ for any $i, j \in \{1, ..., N\}$, we will have $\Delta_{\widetilde{\eta}(\infty)} > 0$, then $\lim_{N\to\infty} C^* > \langle k\rangle$ can be correspondingly obtained. □

A simple realisation of the rule for relaxing C^* is $\lambda_i = 1/k_i$, which applies on both synthetic heterogeneous and empirical networks (Supplementary Figs. 6 and 7). Furthermore, we numerically confirm that the setting of $\lambda_i = 1/w_i$ can promote cooperation on weighted networks (Supplementary Fig. 9).

Supplementary Note 4: General two-player games

In a general two-player game, a cooperator receives "rewards" R from mutual cooperation, while defectors obtain "punishment" P from mutual defection. A defector attempting to exploit a cooperator obtains T and leaves S to its opponent cooperator. The payoff matrix of the game is given by

$$\begin{array}{c c} & \begin{array}{cc} \mathrm{C} & \mathrm{D} \end{array} \\ \begin{array}{c} \mathrm{C} \\ \mathrm{D} \end{array} & \begin{pmatrix} R & S \\ T & P \end{pmatrix} \end{array}.$$

In each round of game, each individual i obtains the average payoff

$$f_i(\mathbf{x}) = (R - S - T + P)x_i \sum_{k=1}^{N} p_{ik}x_k + (S - P)x_i + (T - P)\sum_{k=1}^{N} p_{ik}x_k + P.$$

Following the procedure in the first section, we have

$$\begin{aligned}
\left.\frac{\mathrm{d}\widehat{\Delta}(\mathbf{x})}{\mathrm{d}\delta}\right|_{\delta=0} &= \frac{1}{\Lambda\sum_{i=1}^{N}\frac{w_i}{\lambda_i}}\left(\sum_{i=1}^{N} w_i x_i f_i - \sum_{i,j=1}^{N} w_i p_{ij}^{(2)} x_i f_j\right) \\
&= \frac{1}{\Lambda\sum_{i=1}^{N}\frac{w_i}{\lambda_i}}\left[(R-S-T+P)\left(\sum_{i,j=1}^{N} w_i p_{ij} x_i x_j - \sum_{i,j,k=1}^{N} w_i p_{ij}^{(2)} p_{jk} x_i x_j x_k\right)\right. \\
&+(S-P)\left(\sum_{i=1}^{N} w_i x_i^2 - \sum_{i,j=1}^{N} w_i p_{ij}^{(2)} x_i x_j\right) \\
&\left.+(T-P)\left(\sum_{i,j=1}^{N} w_i p_{ij} x_i x_j - \sum_{i,j=1}^{N} w_i p_{ij}^{(3)} x_i x_j\right)\right],
\end{aligned}$$

By letting the expectation change in reproductive-value-weighted frequency of cooperation under rare-mutation conditional (RMC) distribution $\mathbb{E}_{\mathrm{RMC}}^{\circ}[\left.\frac{\mathrm{d}\widehat{\Delta}(\mathbf{x})}{\mathrm{d}\delta}\right|_{\delta=0}] > 0$, we can obtain the condition under which cooperation is favoured over defection [14], namely $\rho_C > \rho_D$ when

$$(R+S-T-P)\,\tau^{(2)} + (R-S+T-P)\left(\tau^{(3)} - \tau^{(1)}\right) > 0. \tag{22}$$

where the population starts from a uniform initialisation of a single cooperator and $\tau^{(n)} = \sum_{i,j=1}^{N} w_i p_{ij}^{(n)} \tau_{ij}$. Here $\tau_{ij} := \mathbb{E}_{\mathrm{RMC}}^{\circ}\left[\frac{1}{2} - x_i x_j\right]/K^{\circ}$ according to [14]. Note that here $\tau^{(1)}$, $\tau^{(2)}$ and $\tau^{(3)}$ have the exactly same solutions as $\eta^{(1)}$, $\eta^{(2)}$ and $\eta^{(3)}$ in Equation (6).

For a typical prisoner's dilemma where a single parameter $T = b$ is employed to depict the level of social dilemma ($R = 1, S = P = 0$), cooperation is favoured when $b < b^*$. By applying Equation (22), we obtain $b^* = (C^* + 1)/(C^* - 1)$, where C^* is exactly the critical threshold under the donation game in the main text. We show that a higher b^* indicates that cooperation can be promoted even in a high dilemma, which corresponds to a lower C^*. For a typical snowdrift game ($R = \beta - 1/2, S = \beta - 1, T = \beta, P = 0$), we have the critical ratio β^* for promoting the emergence of cooperation when $\beta > \beta^*$. We show that $\beta^* = (3C^* - 1)/(2C^* - 2)$, where β^* decreases when C^* decreases. This implies that when the threshold C^* is lower under the donation game, the threshold β^* for favouring cooperation under snowdrift games is also reduced.

In general, cooperation is favoured over defection when $R > P + (T - S)(C^* - 1)/(C^* + 1)$, where a lower threshold for R can be achieved with a lower C^*. This indicates that the conclusion we obtained under the donation game can be completely applied to different social dilemmas, such as the general prisoner's dilemma ($T > R > P > S$), snowdrift game ($T > R > S > P$) and stag hunt

game ($R > T \geq P > S$). Therefore, the inverse relationship between update rates and node's degree can facilitate the emergence of cooperation in different social dilemmas.

Supplementary Note 5: The optimal strategy update rate for minimising C^*

In order to search the optimal rate ($\lambda_i > 0$) for each node, we set $\lambda_i = \exp(\theta_i)$. Based on the derivation of C^* in Equation (6) and the recurrence relationship in Equation (4), we can calculate $\frac{\partial C^*}{\partial \lambda_i}$ by solving a system of $N(N-1)/2$ linear equations. Thus, to minimise $C^*(\theta_1, \theta_2, ..., \theta_N)$, we develop OptUpRat based on the RMSprop [15] algorithm by calculating the gradient $\left(\frac{\partial C^*}{\partial \theta_1}, \frac{\partial C^*}{\partial \theta_2}, ..., \frac{\partial C^*}{\partial \theta_N}\right)^{\mathrm{T}}$ at each step of iteration. The process starts from $\lambda_i = 1$ for each node, with learning rate of 1, decay rate of 0.9 and ends when $\frac{1}{N}\sum_i |\Delta\lambda_i| < 10^{-5}$. Note that we implement a small random perturbation around $\lambda_i = 1$ on the lattice structure to increase the initial gradient. We present the magnitude of C^* and fixation time during the optimisation procedure in Supplementary Fig. 10. Supplementary Figure 12 shows the convergence of minimising the critical benefit-to-cost ratio C^* on scale-free network and lattice.

Supplementary Note 6: A unifying understanding of the evolution of cooperation on heterogeneous networks

Previous studies have presented conflicting results of the effect of heterogeneous networks on the evolution of cooperation from different theoretical frameworks. The one starting from equal cooperators and defectors takes the measure of the frequency of cooperators, finding that heterogeneous networks promote the emergence of cooperation [16]. Another perspective of fixation probability requires the population to start from a single cooperator and end with full cooperators or defectors. The well-known findings based on this framework indicate that heterogeneous networks impede the emergence of cooperation [1, 12]. Specifically, we recap the results of Fig. 4a in Allen *et al.* [1] and plot the critical benefit-to-cost ratio of scale-free and random regular networks in Supplementary Fig. 13a. We present that the highly heterogeneous scale-free networks require a higher threshold for favouring cooperation than the homogeneous random regular networks over different mean degrees.

Moreover, in another following study by Fotouhi *et al.* [12], the critical ratio C^* is analytically

approximated by

$$C^* \approx \frac{N - 2\langle k^2\rangle/\langle k\rangle^2}{N/\langle k\rangle - 2\langle k^2\rangle/\langle k\rangle^2},$$

where $\langle k\rangle$ is the mean degree and $\langle k^2\rangle$ is the second moment of the degree distribution (equation (4.5) in [12]). For homogeneous networks wherein all nodes have the same degree, the critical ratio degenerates to

$$C^* \approx \frac{N - 2}{N/\langle k\rangle - 2}.$$

In contrast, for heterogeneous networks, $\langle k^2\rangle > \langle k\rangle^2$ since the variance of the degree distribution is always greater than 0, which will lead to a larger critical ratio C^* than homogeneous ones.

We show that the different update rules in those two frameworks lead to the conflict conclusions. Indeed, if we take the death-birth update rule that we used in the main text to calculate the average frequency of cooperators starting from an equal number of cooperators and defectors, we still conclude that heterogeneous networks impede the emergence of cooperation than the homogeneous counterpart (Supplementary Fig. 13b). That is, the update rule determines the conclusions about which structure is more advantageous in promoting the emergence of cooperation.

From the perspective of the microscopic mechanism, we show that the reason why heterogeneous degree distribution promotes cooperation in the studies of Santos *et al.* [16] is that the hubs tend to have infrequent strategy switching. In this series of studies, individual x imitates the strategy of node y with probability $(P_y - P_x)/(k_>(T-S))$ if node y's payoff P_y is higher than that of node x (P_x), and $k_>$ indicates maximum degree of either node. There are two main reasons for the infrequent strategy switching of hubs. The first is that these studies use the accumulated payoff, which naturally leads to the high payoff of hubs and decreases the probability of imitating a neighbour with a higher payoff. The second is that the payoff difference is normalised by $k_>(T-S)$, which is much larger than the payoff difference that a smaller node can have higher than a hub.

We further present a simple example to explain how this update rule is advantageous for the emergence of cooperation in heterogeneous networks (Supplementary Fig. 13c). Here we follow the parameter setting by Santos *et al.* ($T = b, R = 1, P = S = 0$) [16]. The hub node x with k_x neighbours obtains payoff $P_x = 0$ because all its neighbours are defectors, and defective neighbour y accumulates payoff $P_y = k_y b$. Whenever x is updated and y is chosen to be imitated, the probability that x copies the strategy of y is k_y/k_x. Therefore, a cooperative hub still can survive even if all its neighbours are defectors. In contrast, under death-birth update, the probability for x changing to defector is 1. This implies that the update rule applied by Santos *et al.* [16] naturally leads to infrequent updates

of hubs, making it easier for a high-payoff strategy to spread between low-degree nodes than to a hub. And this is consistent with the underlying mechanism studied in the main text, namely when we consider the evolutionary success of cooperators, a low update rate for hubs promotes the evolution of cooperation.

Supplementary Table 1: Statistics of the empirical datasets.

Network statistics	Office	Student	Attiro	San Juan Sur
Number of nodes	92	180	59	75
Number of edges	755	2220	128	155
Mean degree	16.41	24.67	4.34	4.13
Second moment of the degree distribution	326.70	728.81	24.10	21.15
C^* under update rate $\lambda_i = 1$	28.32	36.35	5.25	4.68
C^* under update rate $\lambda_i = 1/k_i$	23.79	30.86	4.57	4.09

The four datasets we employed are collected from the face-to-face contacts between: individuals in an office building (Office) [17], students in 5 classes at a high school (Student) [18], and families in villages (Attiro, San Juan Sur) [19]. When the individual i's update rate λ_i varies inversely with its degree ($\lambda_i = 1/k_i$), the critical ratio C^* decreases—the emergence of cooperation is promoted compared to the scenario with identical update rates ($\lambda_i = 1$).

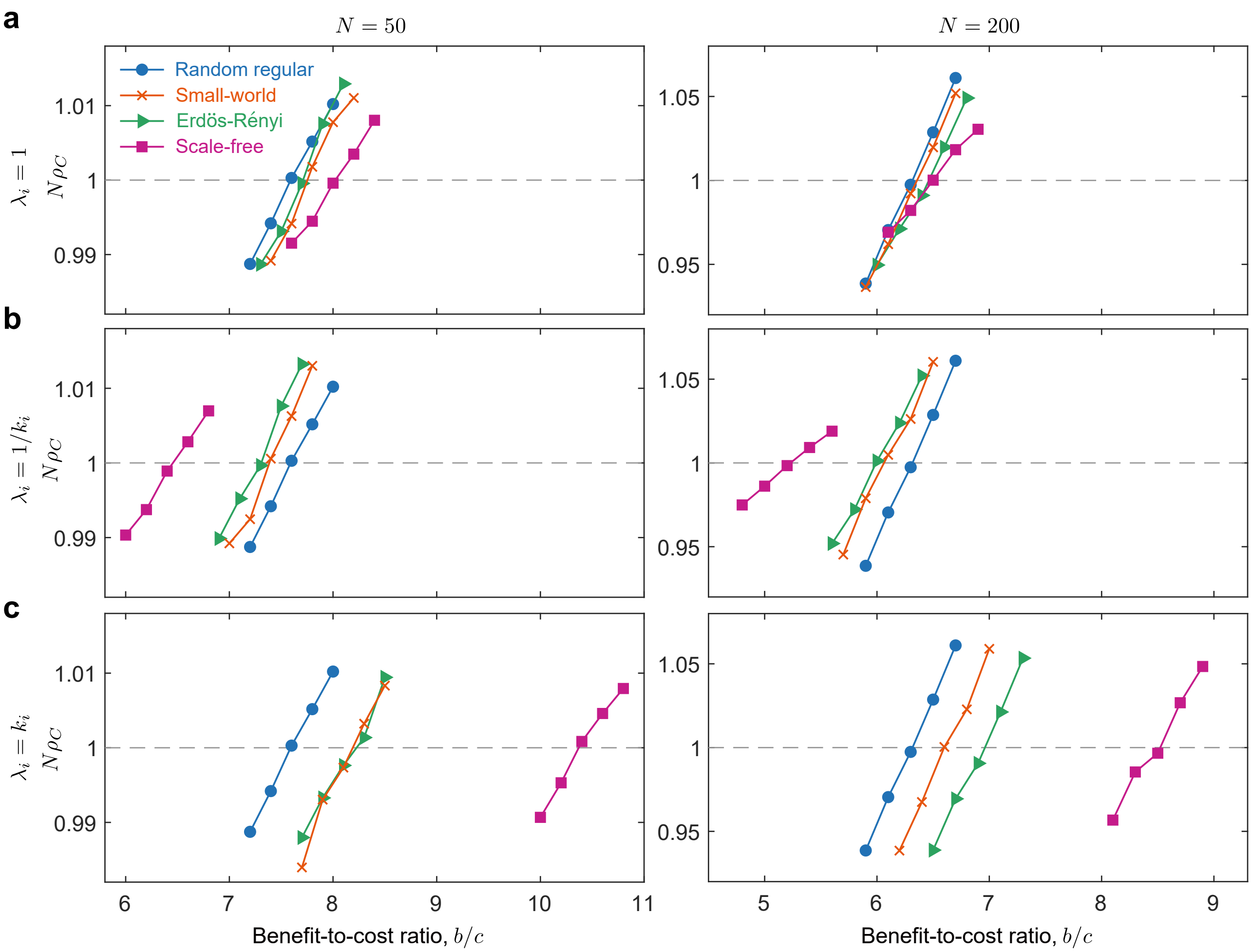

Supplementary Figure 1: Effect of heterogeneous update rates on the evolution of cooperation with different population sizes. We present the fixation probability of cooperation (ρ_C) as a function of the benefit-to-cost ratio (b/c) over identical ($\lambda_i = 1$ for every individual in **a**) and heterogeneous ($\lambda_i = 1/k_i$ in **b**, $\lambda_i = k_i$ in **c**) update rates on random regular, Erdös-Rényi [20], small-world [21] and scale-free [22] networks of $N = 50$ (left panels) and $N = 100$ (right panels), respectively. The critical benefit-to-cost ratio C^* above which the cooperation is favoured for each network occurs when the corresponding curve intersects the horizontal line representing the neutral-drift case ($\rho_C = 1/N$). Other parameters are the same as those in Fig. 2a. We show our results in Fig. 2a are robust with different population size N. Source data are provided as a Source Data file.

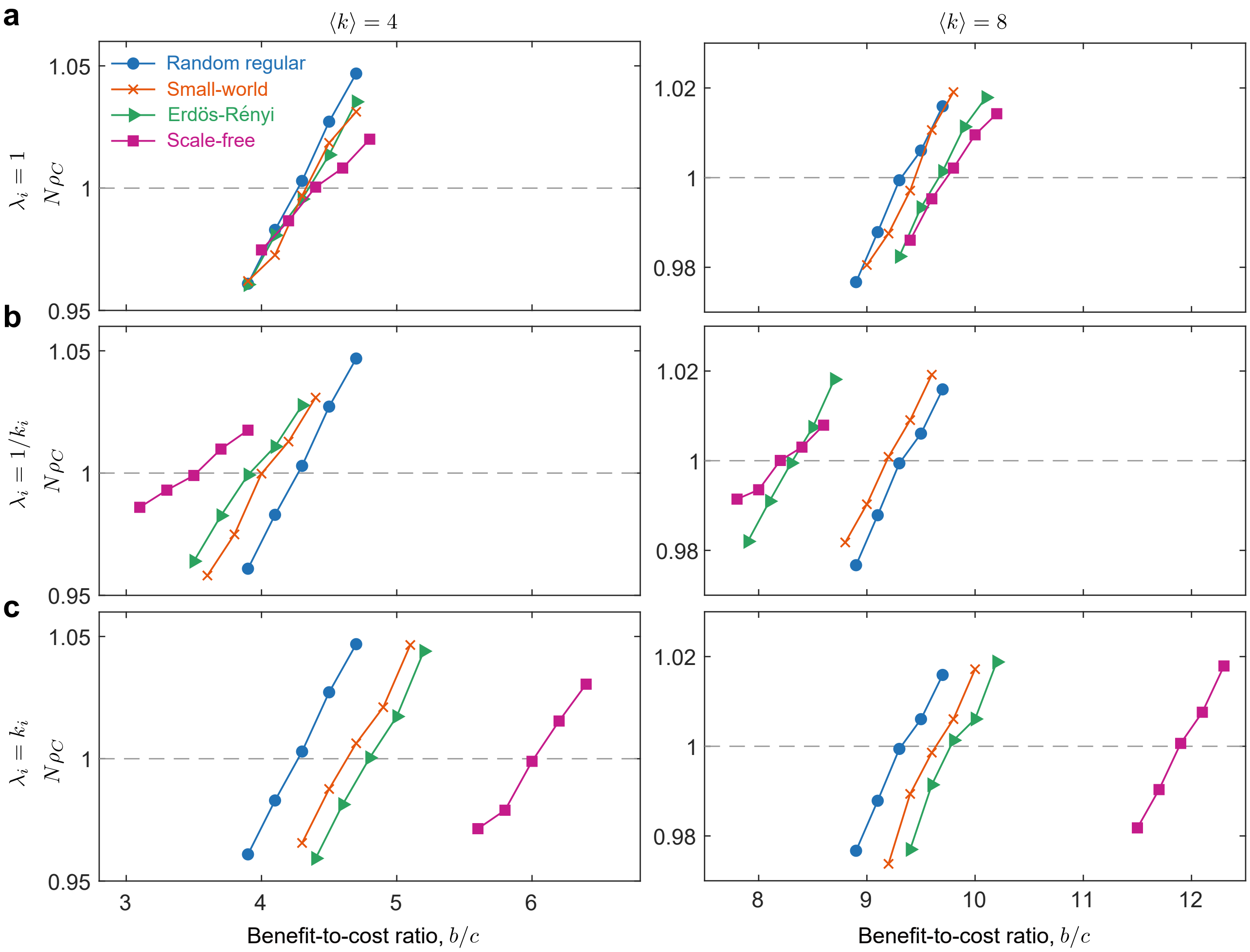

Supplementary Figure 2: Effect of heterogeneous update rates on the evolution of cooperation with different network degrees. We present the fixation probability of cooperation (ρ_C) as a function of the benefit-to-cost ratio (b/c) over identical ($\lambda_i = 1$ for every individual in **a**) and heterogeneous ($\lambda_i = 1/k_i$ in **b**, $\lambda_i = k_i$ in **c**) update rates on random regular, Erdös-Rényi [20], small-world [21] and scale-free [22] networks with average degree $\langle k \rangle = 4$ (left panels) and $\langle k \rangle = 8$ (right panels), respectively. Other parameters are the same as those in Fig. 2a. We show our results in Fig. 2a are robust with different average degree $\langle k \rangle$. Source data are provided as a Source Data file.

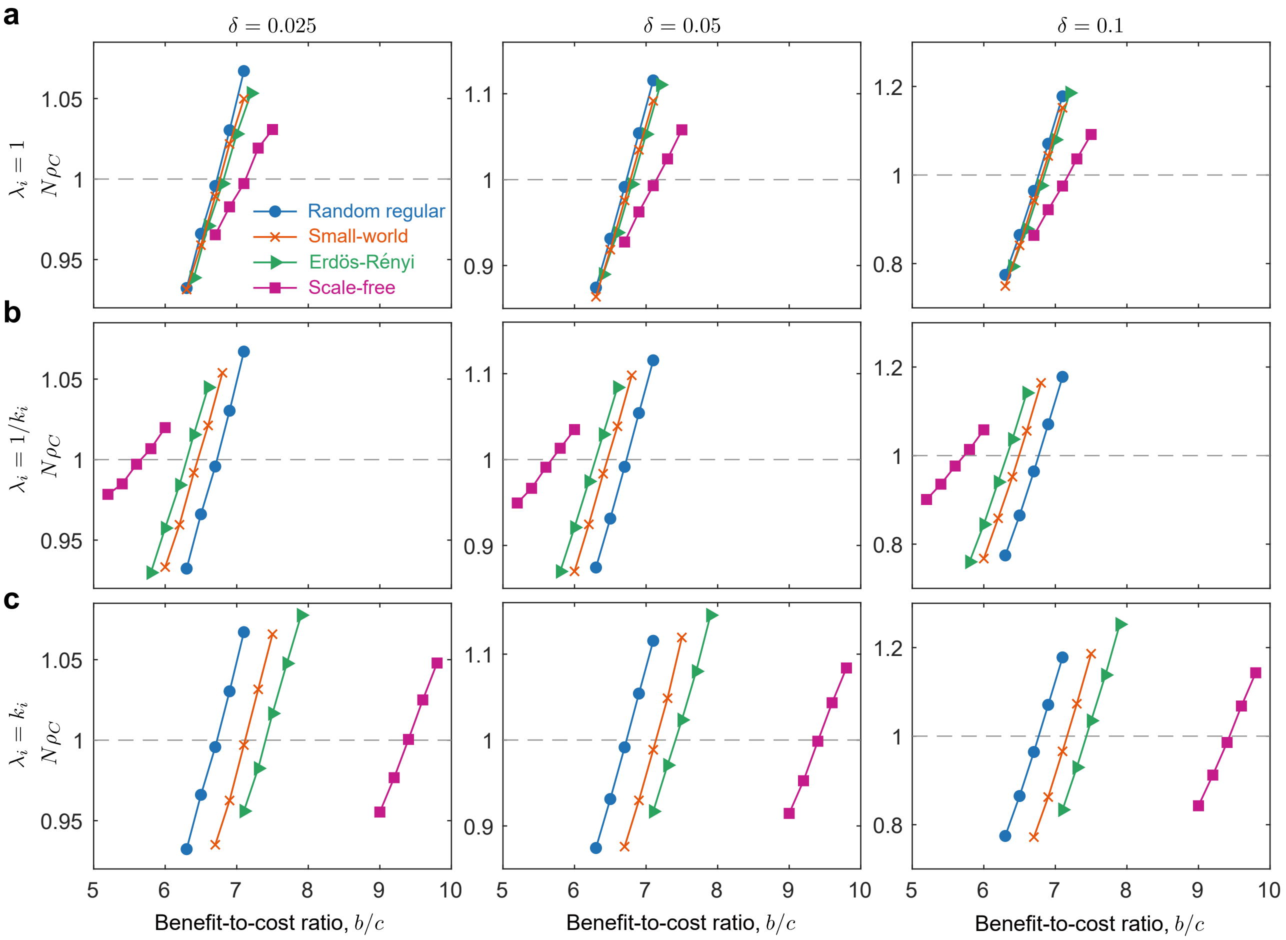

Supplementary Figure 3: Effect of heterogeneous update rates on the evolution of cooperation with different selection intensities. We present the fixation probability of cooperation (ρ_C) as a function of the benefit-to-cost ratio (b/c) over identical ($\lambda_i = 1$ for every individual in **a**) and heterogeneous ($\lambda_i = 1/k_i$ in **b**, $\lambda_i = k_i$ in **c**) update rates on lattice, Erdös-Rényi [20], small-world [21] and scale-free [22] networks under selection intensity $\delta = 0.025$ (left panels), $\delta = 0.05$ (middle panels), and $\delta = 0.1$ (right panels), respectively. Other parameters are the same as those in Fig. 2a. We show our results in Fig. 2a are robust with different selection intensity δ. Source data are provided as a Source Data file.

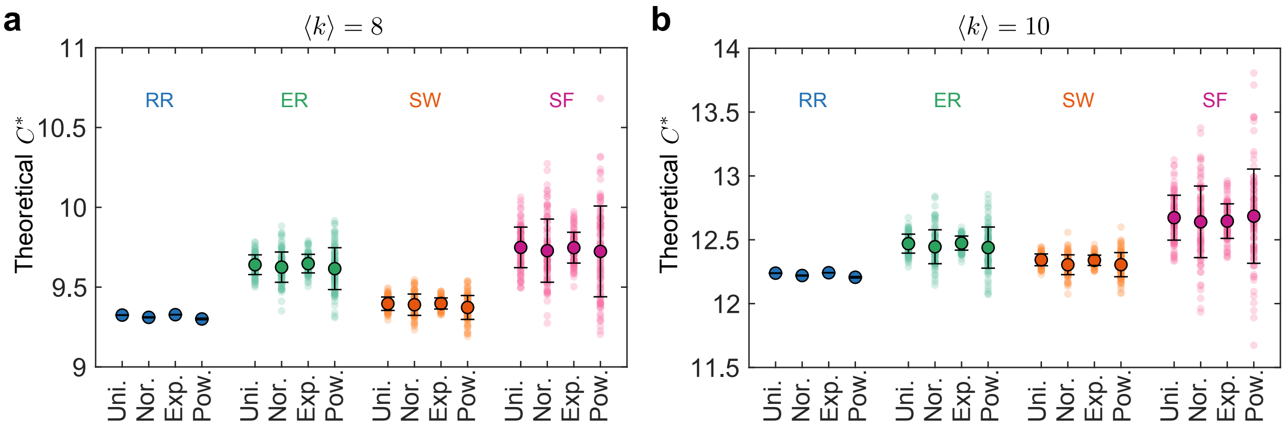

Supplementary Figure 4: Critical benefit-to-cost ratio under different distributions of update rates. We calculate the critical benefit-to-cost ratio C^* under uniform (Uni.), normal (Nor.), exponential (Exp.), and power-law (Pow.) distribution of update rates on random regular (RR), Erdös-Rényi [20] (ER), small-world [21] (SW) and scale-free [22] (SF) networks, respectively. We have the average degree $\langle k \rangle = 8$ of networks in **a** and $\langle k \rangle = 10$ in **b**. Here each dot corresponds to a sample, and the error bars are plotted over 100 samples, indicating the mean values with +/- SD. Other parameters are the same as those in Fig. 6a in the main text. Source data are provided as a Source Data file.

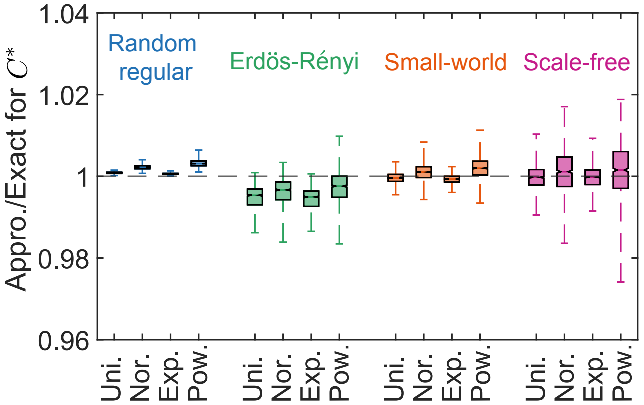

Supplementary Figure 5: Illustration of the accuracy of the approximation of C^* over different distributions of the update rate. We present the ratio between the approximation of C^* calculated from Equation (20) and the exact value obtained from Equation (6), which exhibits the remarkable accuracy over random regular, Erdös-Rényi, small-world and scale-free networks under uniform (Uni.), normal (Nor.), exponential (Exp.) and power-law (Pow.) distributions of update rates. Each box results from realisations of 10 networks and 100 independent update rates. On each box, the central mark indicates the median, and the bottom and top edges of the box indicate the 25th and 75th percentiles, respectively. The whiskers extend to the most extreme data points, and we have no outliers. The uniform distribution is taken from the interval $[0.5, 1.5]$. The normal distribution has the mean of 1 and standard deviation 0.5, where the values of update rate are truncated to be greater than 0. The exponential distribution is with the rate 2 and the power-law distribution is with the exponent -2. The corresponding calculations are shown in Fig. 6a in main text. Source data are provided as a Source Data file.

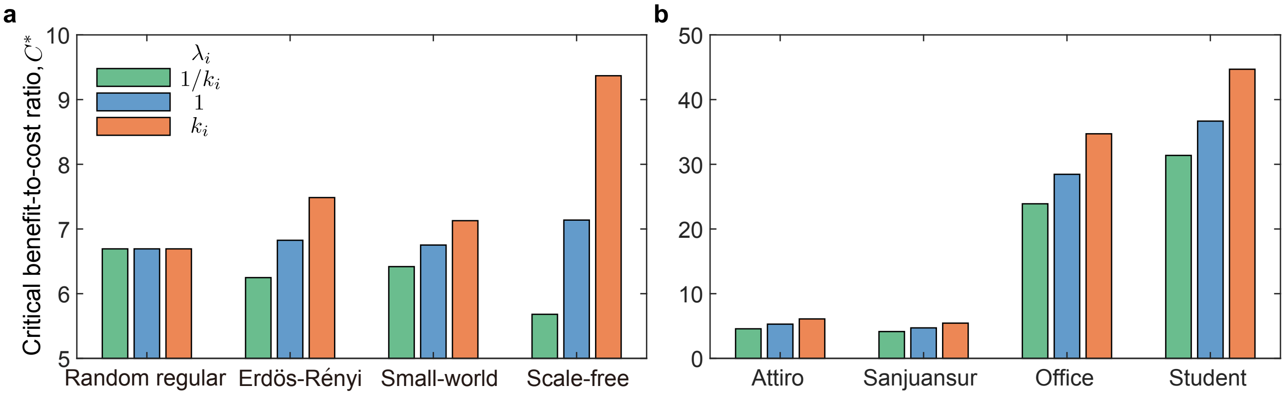

Supplementary Figure 6: The inverse relationship between nodes' degree and individual update rate can relax the critical threshold. We show the critical ratio C^* over different update rates: $\lambda_i = 1/k_i$ (green), $\lambda_i = 1$ (blue) and $\lambda_i = k_i$ (orange). **a**, The setting of update rate with $\lambda_i = 1/k_i$ reduces critical benefit-to-cost ratio C^* for Erdös-Rényi, small-world and scale-free networks compared to identical update rate (blue bar), while update rate $\lambda_i = k_i$ increases C^* for those heterogeneous networks. For random regular networks where $k_i = k$, $\lambda_i = 1/k_i$ and $\lambda_i = k_i$ lead to identical update rate of individuals, thus the critical benefit-to-cost ratio C^* remains same with the case of $\lambda_i = 1$. All of the synthetic networks have the average degree of 6. **b**, The reduction of critical ratio with $\lambda_i = 1/k_i$ is also confirmed on empirical datasets collected from family contacts (Attiro, San Juan Sur) [19], office interactions [17] and student contacts [18]. Numerical values of C^* are obtained from the intersection between fixation probability through benefit-to-cost ratio and the neutral fixation probability (Fig. 2a in main text). And the numerical values of fixation probability are obtained from the fraction of simulations where the population reaches full cooperation out of 10^7 independent runs with $\delta = 0.01$. Source data are provided as a Source Data file.

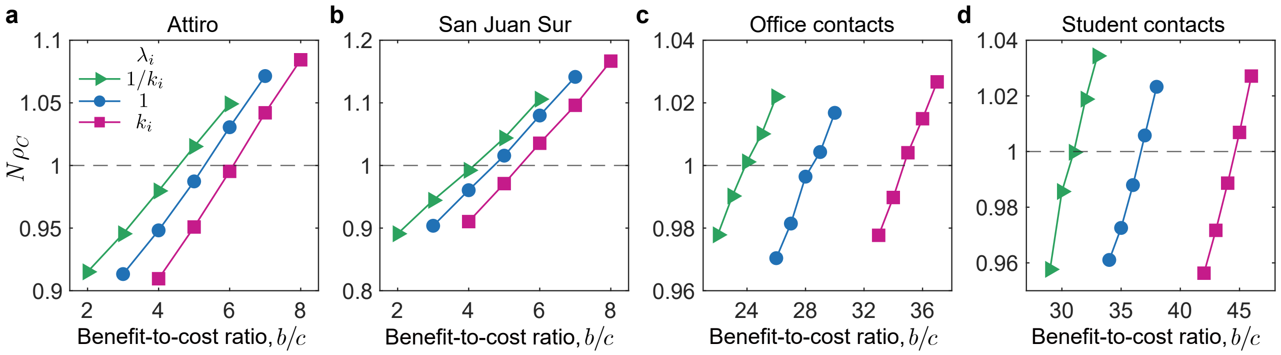

Supplementary Figure 7: The update rate $\lambda_i = 1/k_i$ can promote cooperation on empirical datasets. For four empirical datasets: **a** family contacts (Attiro) [19], **b** family contacts (San Juan Sur) [19], **c** office interactions [17] and **d** student contacts [18], we show the fixation probability of cooperation (ρ_C) through benefit-to-cost ratio (b/c) over update rates $\lambda_i = 1/k_i$, $\lambda_i = 1$, and $\lambda_i = k_i$. All of these datasets show that the fixation probability with update rate $\lambda_i = 1/k_i$ is higher than that with $\lambda_i = 1$, while the case of $\lambda_i = k_i$ decreases the fixation probability compared to that of $\lambda_i = 1$. Source data are provided as a Source Data file.

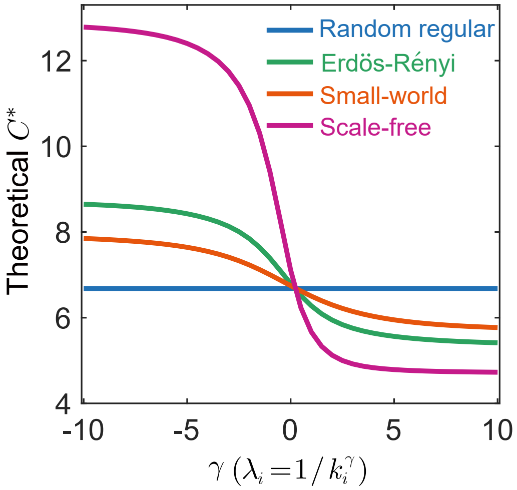

Supplementary Figure 8: Illustration on the variations of C^* when the update rate is correlated with the high-order network degree. We theoretically present the critical benefit-to-cost ratio C^* with update rate $\lambda_i = 1/k_i^\gamma$ on random regular, Erdös-Rényi, small-world and scale-free networks. And C^* decreases when γ increases. Here we set the network size $N = 100$ and average degree $k = 6$ for each network. Source data are provided as a Source Data file.

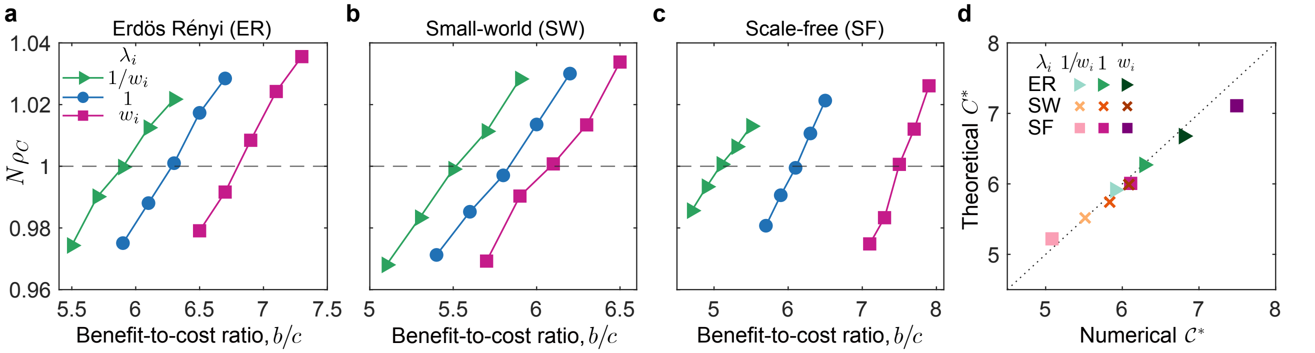

Supplementary Figure 9: The update rate $\lambda_i = 1/w_i$ can promote cooperation on weighted networks. We construct weighted networks by assigning edges on unweighted networks with weights randomly chosen from $\{1, 2, 3\}$. We show the fixation probability of cooperation (ρ_C) through benefit-to-cost ratio (b/c) over different settings of the update rate, namely identical ($\lambda_i = 1$ with blue dots) and heterogeneous ($\lambda_i = 1/w_i$ with green triangles, $\lambda_i = w_i$ with purple squares) rates on weighted Erdös-Rényi (ER), small-world (SW) and scale-free (SF) networks, respectively (**a-c**). **d**, The theoretical results of C^* obtained from our theoretical approximation (Equation (20)) with various update rates (different markers) are in good agreement with numerical simulations on weighted networks. Other settings are the same as those in Fig. 2a in main text. Source data are provided as a Source Data file.

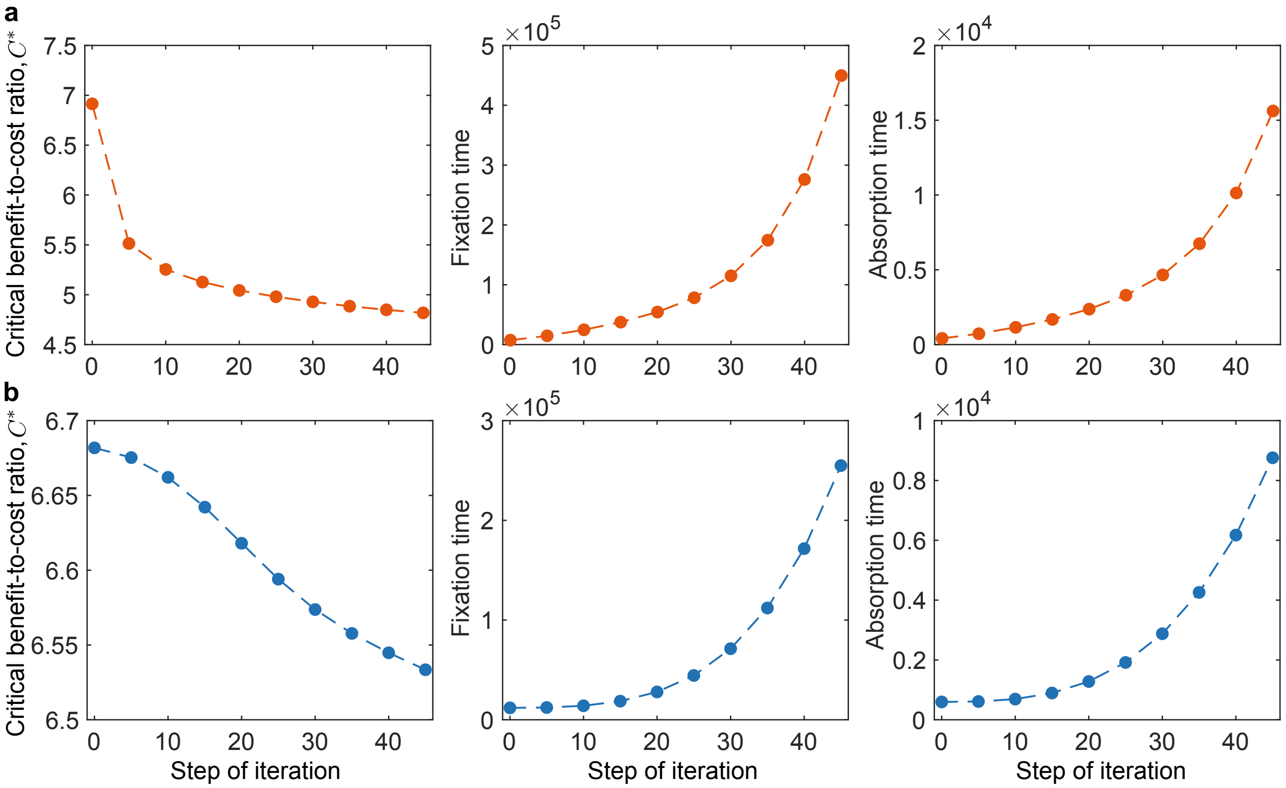

Supplementary Figure 10: The update rate configurations during the optimisation for searching the minimal critical threshold increase the fixation and absorption time. a, The critical ratio C^* decreases through the iteration on a scale-free network, while the fixation time for a random cooperator taking over the whole population also drastically increases. The absorption time describes the average time for all individuals reaching the absorbing state starting from a random cooperator, which is also observed increasing with the reduction of C^* through iteration. The trend of the increase of fixation time and absorption time with the decrease of the critical ratio C^* during the optimisation for searching the minimal critical threshold is also observed on the random regular network (**b**). Both networks have $N = 100$ nodes. Source data are provided as a Source Data file.

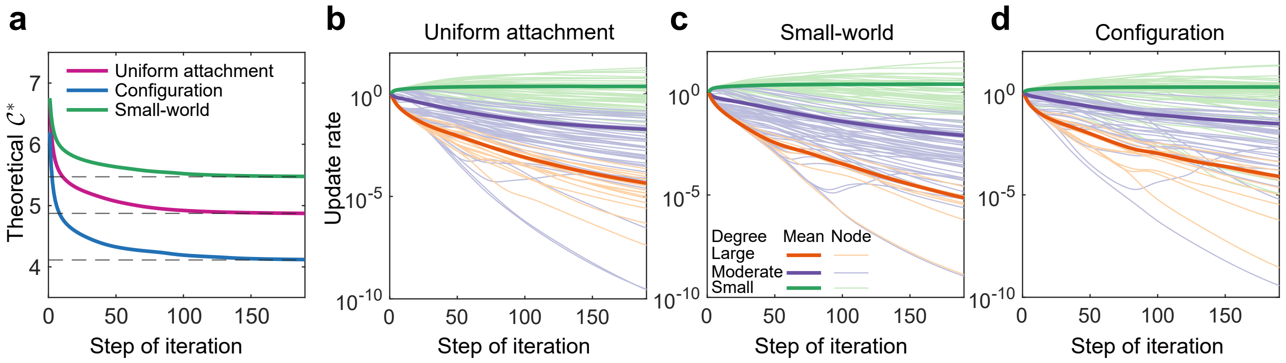

Supplementary Figure 11: Illustration of the process for searching the minimal critical threshold for the emergence of cooperation on different networks. **a**, We present the dynamical evolution of the critical ratio C^* for uniform attachment model, small-world network and configuration model over 190 steps of iterations, which eventually converges to the minimal C^*. We show the evolution of update rates for all nodes separately, which are divided into three categories (large, moderate and small), based on the number of their neighbours (node degree) for uniform attachment model (**b**), small-world network (with rewiring probability 0.7) (**c**) and configuration model (**d**). The mean update rates in each category are presented with the corresponding thicker line. The optimal update rates decrease for large nodes and increase for small nodes generally on all these three networks. Each network has $N = 100$ nodes. Source data are provided as a Source Data file.

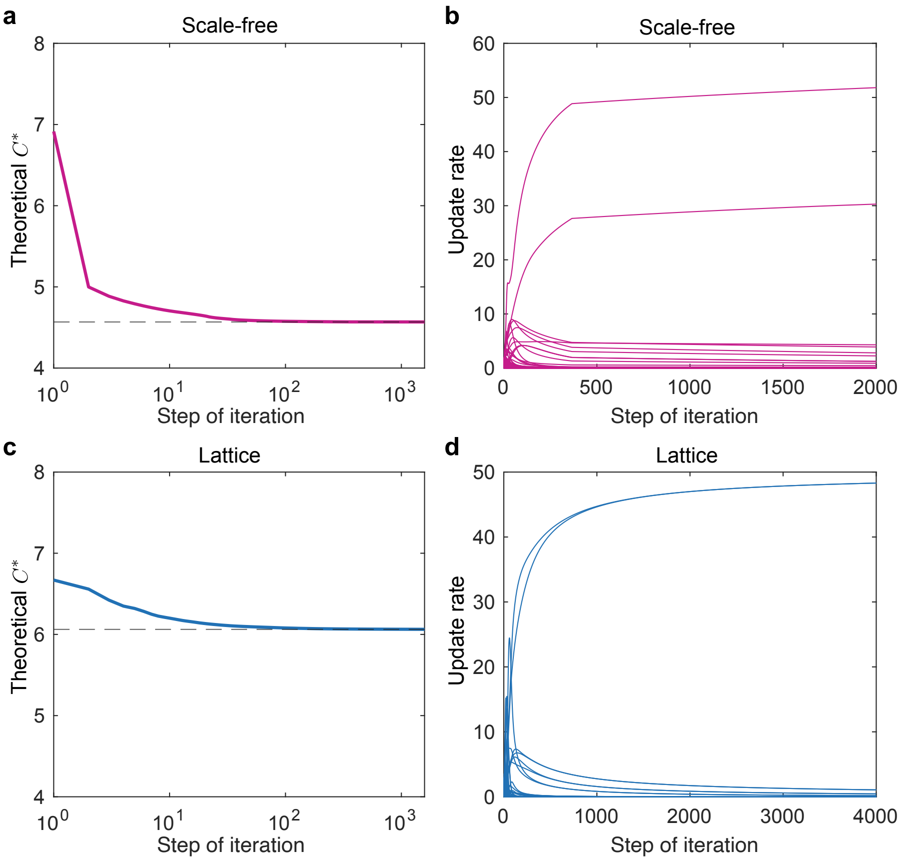

Supplementary Figure 12: Illustration of the process for searching the minimal critical threshold for the emergence of cooperation on scale-free network and lattice. We present the dynamical evolution of the critical ratio C^* for scale-free network and lattice in **a** and **c**. And we also show that the update rates of both networks converge through the iterations (**b**, **d**). Other settings are the same as those in Fig. 7d in main text. Source data are provided as a Source Data file.

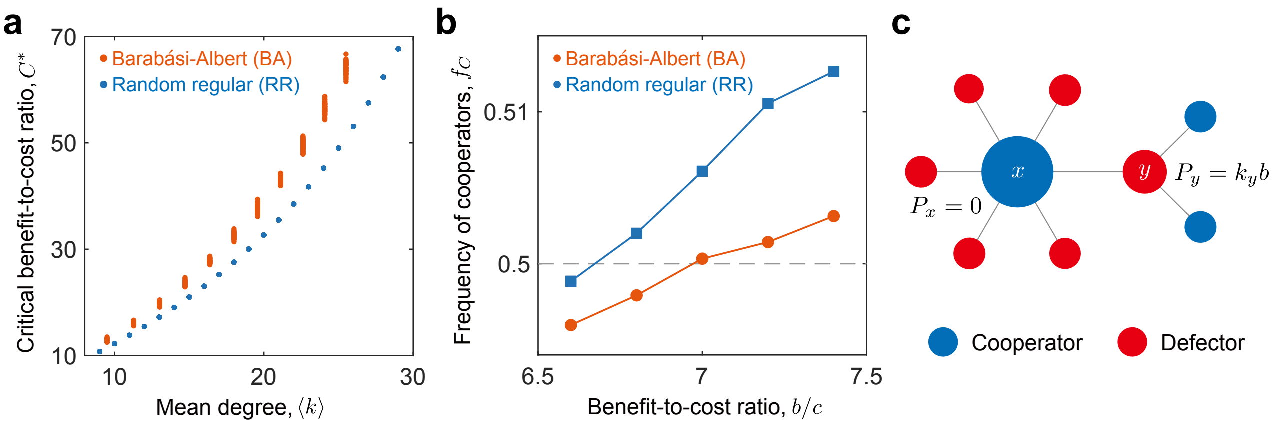

Supplementary Figure 13: Effect of different update rules on the evolution of cooperation. **a**, Scatter plot of the critical benefit-to-cost ratio C^* versus mean degree $\langle k \rangle$ of scale-free (orange dot) and random regular (blue dot) networks with $N = 100$. **b**, Regarding the frequency of cooperators in the population starting from equal cooperators and defectors [16], scale-free networks (orange) lead to a lower frequency of cooperation compared to random regular networks (blue) under death-birth update. **c**, The cooperator x (blue) with all defective neighbours (red) obtains $P_x = 0$, and the defector y with k_y cooperative neighbours obtains $P_y = k_y b$. Under the update rule in the series of studies by Santos *et al.*, whenever x is updated and y is randomly chosen to be imitated, individual x imitates the strategy of y with probability k_y/k_x. Source data are provided as a Source Data file.

Supplementary References

[1] Allen, B., Lippner, G., Chen, Y.-T., Fotouhi, B., Momeni, N., Yau, S.-T. & Nowak, M. A. Evolutionary dynamics on any population structure. *Nature* **544**, 227–230 (2017).

[2] Hilbe, C., Nowak, M. A. & Sigmund, K. Evolution of extortion in iterated prisoner's dilemma games. *Proc. Natl Acad. Sci. USA* **110**, 6913–6918 (2013).

[3] Fisher, R. A. *The Genetical Theory of Natural Selection* (Clarendon Press, 1930).

[4] Taylor, P. D. Allele-Frequency Change in a Class-Structured Population. *Am. Nat.* **135**, 95–106 (1990).

[5] Allen, B. & McAvoy, A. A mathematical formalism for natural selection with arbitrary spatial and genetic structure. *J. Math. Biol.* **78**, 1147–1210 (2019).

[6] McAvoy, A. & Allen, B. Fixation probabilities in evolutionary dynamics under weak selection. *J. Math. Biol.* **82**, 1–41 (2021).

[7] Tarnita, C. E. & Taylor, P. D. Measures of relative fitness of social behaviors in finite structured population models. *Am. Nat.* **184**, 477–488 (2014).

[8] Kingman, J. F. C. The coalescent. *Stochastic Process. Appl.* **13**, 235–248 (1982).

[9] Cox, J. T. Coalescing random walks and voter model consensus times on the torus in $\mathbf{Z}^{\mathrm{d}}$. *Ann. Probab.* **17**, 1333–1366 (1989).

[10] Liggett, T. M. & Liggett, T. M. *Interacting Particle Systems* (Springer, 1985).

[11] Wakeley, J. *Coalescent Theory: an Introduction* (Roberts and Company Publishers, 2009).

[12] Fotouhi, B., Momeni, N., Allen, B. & Nowak, M. A. Evolution of cooperation on large networks with community structure. *J. R. Soc. Interface* **16**, 20180677 (2019).

[13] Ohtsuki, H., Hauert, C., Lieberman, E. & Nowak, M. A. A simple rule for the evolution of cooperation on graphs and social networks. *Nature* **441**, 502–505 (2006).

[14] McAvoy, A., Allen, B. & Nowak, M. A. Social goods dilemmas in heterogeneous societies. *Nat. Hum. Behav.* **4**, 819–831 (2020).

[15] Tieleman, T. & Hinton, G. Lecture 6.5-rmsprop: Divide the gradient by a running average of its recent magnitude. *COURSERA: Neural Networks for Machine Learning* (2012).

[16] Santos, F. C. & Pacheco, J. M. Scale-free networks provide a unifying framework for the emergence of cooperation. *Phys. Rev. Lett.* **95**, 098104 (2005).

[17] Génois, M., Vestergaard, C. L., Fournet, J., Panisson, A., Bonmarin, I. & Barrat, A. Data on face-to-face contacts in an office building suggest a low-cost vaccination strategy based on community linkers. *Netw. Sci.* **3**, 326–347 (2015).

[18] Fournet, J. & Barrat, A. Contact patterns among high school students. *PLoS One* **9**, e107878 (2014).

[19] de Nooy, W., Mrvar, A. & Batagelj, V. *Exploratory Social Network Analysis with Pajek* (Cambridge Univ. Press, 2005).

[20] Erdös, P. & Rényi, A. On random graphs I. *Publ. Math. (Debrecen)* **6**, 290–297 (1959).

[21] Watts, D. J. & Strogatz, S. H. Collective dynamics of 'small-world'networks. *Nature* **393**, 440–442 (1998).

[22] Barabási, A.-L. & Albert, R. Emergence of scaling in random networks. *Science* **286**, 509–512 (1999).
