# Supplementary material for: Dynamics of collective cooperation under personalised strategy updates
Source: Nat Commun. 2024 Apr 11;15:3125. doi: 10.1038/s41467-024-47380-8 (PMC11006938; doi:10.1038/s41467-024-47380-8)
Supplement: Supplementary file 3 — Reporting Summary [file 41467_2024_47380_MOESM3_ESM.pdf]

nature portfolio

Corresponding author(s): Aming Li

Last updated by author(s): Mar 19, 2024

Reporting Summary

Nature Portfolio wishes to improve the reproducibility of the work that we publish. This form provides structure for consistency and transparency in reporting. For further information on Nature Portfolio policies, see our Editorial Policies and the Editorial Policy Checklist.

Statistics

For all statistical analyses, confirm that the following items are present in the figure legend, table legend, main text, or Methods section.

n/a	Confirmed	
☐	☒	The exact sample size (*n*) for each experimental group/condition, given as a discrete number and unit of measurement
☐	☒	A statement on whether measurements were taken from distinct samples or whether the same sample was measured repeatedly
☒	☐	The statistical test(s) used AND whether they are one- or two-sided<br>*Only common tests should be described solely by name; describe more complex techniques in the Methods section.*
☒	☐	A description of all covariates tested
☒	☐	A description of any assumptions or corrections, such as tests of normality and adjustment for multiple comparisons
☐	☒	A full description of the statistical parameters including central tendency (e.g. means) or other basic estimates (e.g. regression coefficient) AND variation (e.g. standard deviation) or associated estimates of uncertainty (e.g. confidence intervals)
☒	☐	For null hypothesis testing, the test statistic (e.g. *F*, *t*, *r*) with confidence intervals, effect sizes, degrees of freedom and *P* value noted<br>*Give P values as exact values whenever suitable.*
☒	☐	For Bayesian analysis, information on the choice of priors and Markov chain Monte Carlo settings
☒	☐	For hierarchical and complex designs, identification of the appropriate level for tests and full reporting of outcomes
☒	☐	Estimates of effect sizes (e.g. Cohen's *d*, Pearson's *r*), indicating how they were calculated

Our web collection on statistics for biologists contains articles on many of the points above.

Software and code

Policy information about availability of computer code

Data collection	Data was collected using Python code (Version 3.8.5).
Data analysis	Data was analysed using MathWorks MATLAB code (Version R2021a). Source codes can be found at https://github.com/yaomeng1/PersonalizedStrategyUpdates.

For manuscripts utilizing custom algorithms or software that are central to the research but not yet described in published literature, software must be made available to editors and reviewers. We strongly encourage code deposition in a community repository (e.g. GitHub). See the Nature Portfolio guidelines for submitting code & software for further information.

Data

Policy information about availability of data

All manuscripts must include a data availability statement. This statement should provide the following information, where applicable:

- Accession codes, unique identifiers, or web links for publicly available datasets
- A description of any restrictions on data availability
- For clinical datasets or third party data, please ensure that the statement adheres to our policy

Source data are provided as a Source Data file. Data of empirical networks analysed in Fig. 6b are publicly available and can be found in the corresponding references [51, 52].

Research involving human participants, their data, or biological material

Policy information about studies with human participants or human data. See also policy information about sex, gender (identity/presentation), and sexual orientation and race, ethnicity and racism.

Reporting on sex and gender	n/a
Reporting on race, ethnicity, or other socially relevant groupings	n/a
Population characteristics	n/a
Recruitment	n/a
Ethics oversight	n/a

Note that full information on the approval of the study protocol must also be provided in the manuscript.

Field-specific reporting

Please select the one below that is the best fit for your research. If you are not sure, read the appropriate sections before making your selection.

☐ Life sciences ☐ Behavioural & social sciences ☒ Ecological, evolutionary & environmental sciences

For a reference copy of the document with all sections, see nature.com/documents/nr-reporting-summary-flat.pdf

Ecological, evolutionary & environmental sciences study design

All studies must disclose on these points even when the disclosure is negative.

Study description	We studied the condition for strategy success in evolutionary games on networks with arbitrary strategy updates. We generated the synthetic network structures using Python to depict the structured populations. Our results were based on mathematical derivations, proofs (see details in the Supplementary Information) and confirmed with theoretical calculations (100 samples of update rates from each distribution) and numerical Monte Carlo simulations (10^7 independent realizations on each network (n=100) and setting of update rates).
Research sample	For the numerical simulations, fixation probability was obtained from the fraction of simulations in which the population (n=100) reaches full cooperation out of 10^7 independent realisations, with a total number of simulation time steps to be 10^8 to ensure that the population reaches full cooperation or full defection. The structures of populations are defined by synthetic or empirical networks. For theoretical calculations, we took 100 samples of update rates from uniform, normal, exponential and power law distributions on each network structure independently.
Sampling strategy	We considered four different distributions (uniform, normal, exponential and power law) on different network structures. For each network, we randomly took 100 samples of update rates from each distribution independently and calculated the corresponding critical ratio. The sample size is sufficient to observe the difference of C* on different networks for all distributions (Fig. 6a).
Data collection	Data was generated from numerical simulations using Python code and recorded by Y.M..
Timing and spatial scale	Not applicable.
Data exclusions	Not applicable.
Reproducibility	Our results can be reproduced using the code provided in the code availability statement on any given network structures.
Randomization	Not applicable.
Blinding	No blinding was needed for the computer-generated data.

Did the study involve field work? ☐ Yes ☒ No

Reporting for specific materials, systems and methods

We require information from authors about some types of materials, experimental systems and methods used in many studies. Here, indicate whether each material, system or method listed is relevant to your study. If you are not sure if a list item applies to your research, read the appropriate section before selecting a response.

Materials & experimental systems

n/a	Involved in the study
☒	☐ Antibodies
☒	☐ Eukaryotic cell lines
☒	☐ Palaeontology and archaeology
☒	☐ Animals and other organisms
☒	☐ Clinical data
☒	☐ Dual use research of concern
☒	☐ Plants

Methods

n/a	Involved in the study
☒	☐ ChIP-seq
☒	☐ Flow cytometry
☒	☐ MRI-based neuroimaging

Plants

Seed stocks	n/a
Novel plant genotypes	n/a
Authentication	n/a
